# Fabrication of SWCNT-Ag Nanoparticle Hybrid Included Self-Assemblies for Antibacterial Applications

**DOI:** 10.1371/journal.pone.0106775

**Published:** 2014-09-05

**Authors:** Sayanti Brahmachari, Subhra Kanti Mandal, Prasanta Kumar Das

**Affiliations:** Department of Biological Chemistry, Indian Association for the Cultivation of Science, Kolkata, India; RMIT University, Australia

## Abstract

The present article reports the development of soft nanohybrids comprising of single walled carbon nanotube (SWCNT) included silver nanoparticles (AgNPs) having superior antibacterial property. In this regard aqueous dispersing agent of carbon nanotube (CNT) containing a silver ion reducing unit was synthesised by the inclusion of tryptophan and tyrosine within the backbone of the amphiphile. The dispersions were characterized spectroscopically and microscopically using TEM, AFM and Raman spectroscopy. The nanotube-nanoparticle conjugates were prepared by the *in situ* photoreduction of AgNO_3_. The phenolate residue and the indole moieties of tyrosine and tryptophan, respectively reduces the sliver ion as well as acts as stabilizing agents for the synthesized AgNPs. The nanohybrids were characterized using TEM and AFM. The antibacterial activity of the nanohybrids was studied against Gram-positive (*Bacillus subtilis* and *Micrococcus luteus*) and Gram-negative bacteria (*Escherichia coli* and *Klebsiella aerogenes*). The SWCNT dispersions showed moderate killing ability (40–60%) against Gram-positive bacteria however no antibacterial activity was observed against the Gram negative ones. Interestingly, the developed SWCNT-amphiphile-AgNP nanohybrids exhibited significant killing ability (∼90%) against all bacteria. Importantly, the cell viability of these newly developed self-assemblies was checked towards chinese hamster ovarian cells and high cell viability was observed after 24 h of incubation. This specific killing of bacterial cells may have been achieved due to the presence of higher –SH containing proteins in the cell walls of the bacteria. The developed nanohybrids were subsequently infused into tissue engineering scaffold agar-gelatin films and the films similarly showed bactericidal activity towards both kinds of bacterial strains while allowing normal growth of eukaryotic cells on the surface of the films.

## Introduction

Over the decades, the development of novel antimicrobial agents has undergone a continuous process of evolution and still remains an important domain of research [1]–[13]. The growing resistance of microbes against the conventional antibiotics necessitated the restructuring of the antibiotic design and newer formulations have emerged with time. This drug resistance mostly arises as a natural process of adaptation and random selection through mutation. To this end, in addition to the conventionally known antibiotics, nanomaterials like silver nanoparticles (AgNPs) have emerged as a class of alternative antibiotics possessing a different mechanism of bacteria killing [14]–[19]. The mechanism of antibacterial activity of AgNPs is still not well understood, however there are theories like (i) membrane damage by free radicals, (ii) membrane structure degradation by “pits” in cell walls, and (iii) penetration of cell walls and dephosphorylation of key peptides in cellular signalling cycles [20]–[22]. To date there are few reports of bacterial resistance towards these nanoparticles and this antibacterial activity has enhanced the broad spectrum applications of these nanomaterials [23]–[24]. In fact recently gold and silver nanoparticles were shown to have specific antibacterial and anticancer activities and AgNP is being used to incorporate antimicrobial activity in paints and biomedical implants [20], [25]–[27].

Single walled carbon nanotube (SWCNTs) – the one dimensional allotrope of carbon, also belongs to the important class of nanomaterials because of its extraordinary optical, electronic, mechanical properties and high aspect ratio [28]–[31]. It is finding applications in almost all branches of sciences from energy research to biotechnology due to its unique intrinsic features. Amongst others, the huge surface area of carbon nanotubes (CNTs) makes it suitable to be utilized as cellular transporters [28]–[31]. However, studies on its interaction with the prokaryotic cells have received comparatively little attention. Few recent reports investigated the antibacterial activity of CNTs and its modified forms [32]–[41]. Size dependent antibacterial activity of CNTs was first reported by Kang et al [33]. Later on the findings were further supported by Liu et al where the membrane damage of prokaryotic cells resulting from direct contact with pristine SWCNTs was investigated [36]. However, the inherent insolubility of these nanostructures greatly bars its applications and the key towards exploiting this nanomaterial in the biomedicinal arena lies in designing judicious CNT dispersing agents [42]–[51]. To this end, we recently reported amino acid based biocompatible SWCNT dispersions for the delivery of biomolecules and drugs into eukaryotic cells [52]–[55]. Additionally we have also utilized AgNPs for the development of composite hydrogel matrices having antibacterial activity [56]–[58]. However it would be intriguing if the complementary properties of these two nanomaterials could be simultaneously exploited to develop superior antimicrobial agents. In particular, fabrication of AgNPs decorated CNT dissolution in aqueous medium by the assistance of amphiphilic dispersing agent could be used for developing antibacterial scaffolds. Such soft-nanocomposites would find wide range application in the biomedicinal arena including tissue engineering, drug delivery and so forth.

**Figure 1:**
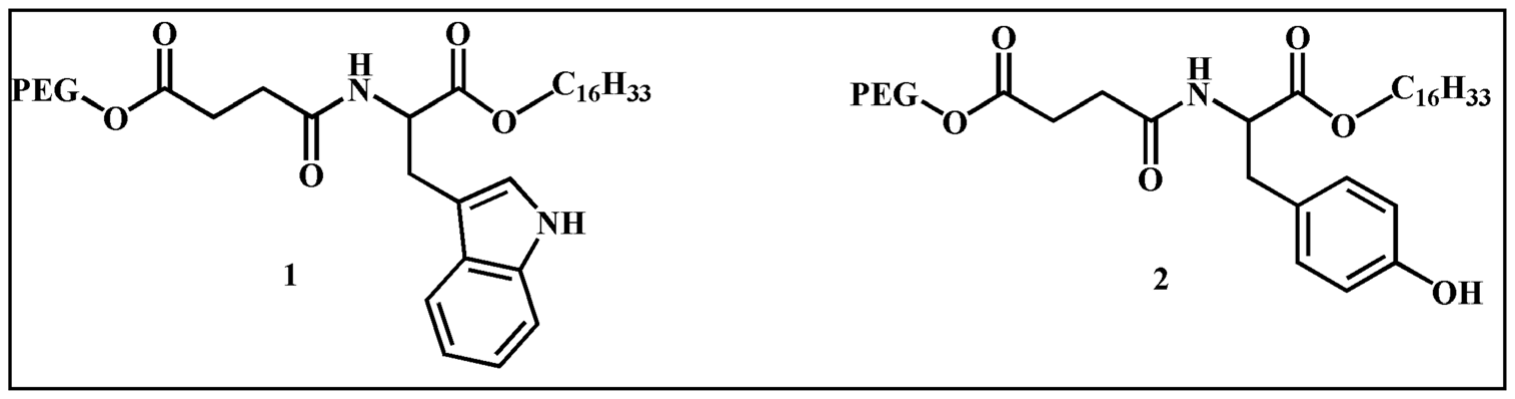
Structure of the dispersing agents. PEG =  -(CH_2_CH_2_O)_12_CH_3_.

Herein, the present work reports the design and development of aqueous SWCNT dispersion by L-tyrosine and L-tryptophan based neutral amphiphiles (Figure 1) comprising of polyethylene glycol (PEG) unit. The AgNP was synthesized within these dispersions by *in situ* photo-reduction under sunlight [59]. The SWCNT dispersion and nanoconjugates were characterized using transmission elector microscopy (TEM), atomic force microscopy (AFM) and Raman spectroscopy. Encouragingly, more than 90% killing of both Gram-positive (*Bacillus subtilis* and *Micrococcus luteus*) and Gram-negative bacteria (*Escherichia coli* and *Klebsiella aerogenes*) was achieved using SWCNT-amphiphile-AgNP hybrid (6–10 µg/mL AgNP). Moreover, substantial cell viability of the nanohybrids was observed against Chinese Hamster Ovarian cells (CHO cells). Interestingly, normal growth of eukaryotic cells were noted on the surface of these nanocomposites infused agar-gelatin film (tissue engineering scaffold) while it was lethal toward bacteria.

## Results and Discussion

### Synthesis of dispersing agent

Development of SWCNT based antimicrobial agents is still at its infancy while its potential demands wider exploitation particularly in the backdrop of antibiotics resistance microbes. The first step towards developing biocompatible antibacterial dispersion of CNTs is to design dispersing agents that would facilitate the exfoliation of CNTs in water. Any non-covalent SWCNT dispersing agent typically contains a hydrophobic and a hydrophilic end. Generally, the hydrophobic unit binds to the surface of the nanotubes while the hydrophilic end assists its solubilization in water through the formation of supramolecular aggregates. To this end recently we reported the formation of electrostatically bound composite material of SWCNT and gold nanoparticles where the nanotubes were dispersed in water using cationic amphiphile and the nanoparticles were capped with anionic surfactants [60]. However in several previous instances the cationic dispersing agents was found to be cytotoxic towards mammalian cells and hence those SWCNT dispersions were not suitable for developing biocompatible scaffolds [54], [56]. On the other hand, nanotubes dispersing with anionic surfactants may result in overall repulsion with the negatively charged membrane of prokaryotic and eukaryotic cells. Hence, instead of cationic or anionic hydrophilic head groups, we designed amino acid based neutral dispersing agent comprising of polyethylene glycol (PEG) as the hydrophilic unit. The cetyl (C-16) chain generally acts as a good surface anchoring unit for the CNTs and it was taken as the hydrophobic unit. Additionally, the presence of a nanoparticle capping residue was mandatory to ensure the binding of AgNP on the nanotube surface. Therefore, tryptophan and tyrosine amino acids were integrated into the backbone of dispersing agent because of the well known capping and nanoparticle stabilizing ability of the indole residue of tryptophan and phenolate residue of tyrosine [56]–[58]. Thus, amphiphilic dispersing gents **1** and **2** were synthesized by coupling of the C-16 chain to the acid terminal of the amino acid and PEG to the amine terminal via a succinic acid linker (Figure 1).

### Quantification and characterization of SWCNT-dispersions

Aqueous suspensions of SWCNT using **1** and **2** were prepared following the previously reported protocol [52]. Briefly, SWCNT (1 mg) was taken in 4 mL of amphiphile solution (2.5 mg/mL) and tip sonicated followed by bath sonication. The suspension was then centrifuged at 2500 g for 90 min. The supernatant was collected and the amount of dispersed SWCNT was calculated using the previously reported calibration plot prepared using commercially available surfactant sodium dodecyl benzene sulfonate (SDBS) [53]. Both neutral amphiphiles dispersed SWCNTs with an efficient of 75% and 78%, respectively for **1** and **2**, which indicates most of the nanotubes remain in the suspension with respect to its initial weight.

**Figure 2:**
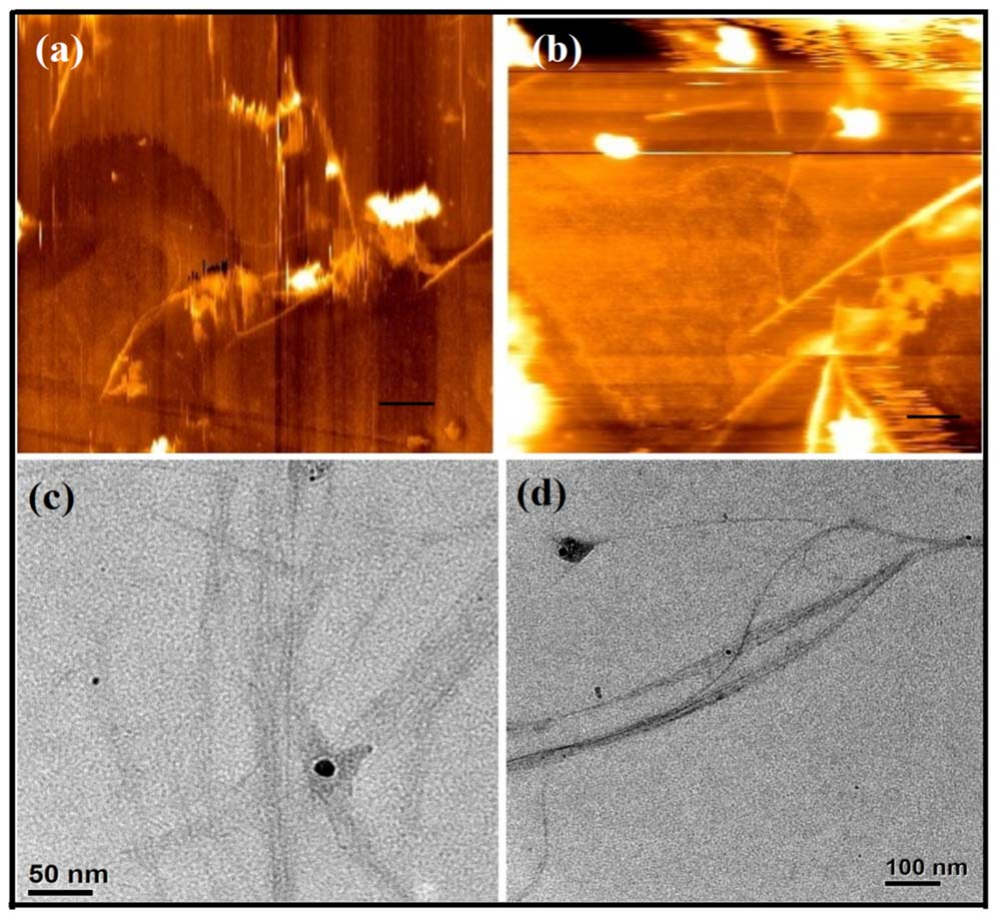
AFM images of dispersed (a) SWCNT-1 and (b) SWCNT-2 (the scale bars in the AFM images indicate 200 nm) and TEM images of dispersed (c) SWCNT-1 (d) SWCNT-2.

These aqueous SWCNT suspensions (SWCNT-**1** and SWCNT-**2**) were stable for months and showed no sign of aggregation/precipitation. In case of positively/negatively charged dispersing agents, the electrostatic repulsion between the nanotubes hindered their re-aggregation while in case of dispersion with neutral PEG based amphiphiles, the steric bulk of the PEG facilitated the dispersion of the nanotubes. Also the hydrogen-bonded water molecules with glycol chain further aided dissolution of SWCNT possibly by preventing the coagulation of nanotubes [61]. These SWCNT dispersions were characterized by microscopic and spectroscopic analysis. In accordance to the atomic force microscopy (AFM) and transmission electron microscopy (TEM) images the diameter of debundled SWCNT-**1** and SWCNT-**2** was found to be ∼ 5 nm (Figure 2). Statistical AFM analysis was performed in order to compare the dimension of individualized nanotubes in an aqueous suspension of **1** and **2**. From several AFM images the average bundle diameter and length of the exfoliated nanotubes were found to between 4–6 nm and 400–500 nm, respectively (Figure S1, S2).

**Figure 3:**
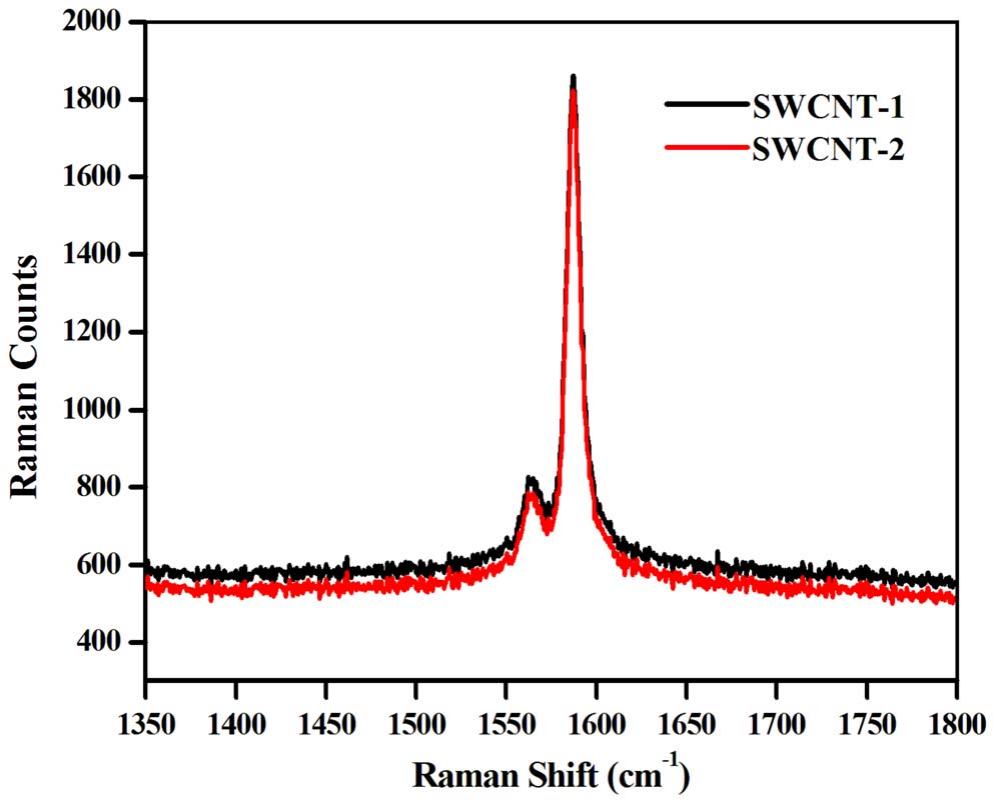
Raman Spectra of dispersed SWCNT-1 and SWCNT-2 using 514.5 nm excitation.

Zeta (*ζ*)-potential is another well established parameter of assessing the colloidal stability of any dispersion [52], [61]. It gives an idea about the interplay of the different forces operating to inhibit the aggregation of the nanomaterials. Conventionally, *ζ*-potential values higher and lower than +/−15 mV indicate greater stability of a colloidal dispersion. In the present study, the *ζ*-potential values were found to be −23 mV and −30 mV, respectively for the aqueous suspension of SWCNT-**1** and SWCNT-**2** [61]. Considering the neutral nature of dispersing agents, the high *ζ*-potential value clearly indicates substantial stability of these SWCNT dispersions. The quality of nanotube dispersions was also studied using Raman spectroscopy. Primarily, the G-band of SWCNT originates from several tangential C–C stretching transitions of the SWCNT carbon atoms whereas the D-band is generally associated with defects in the SWCNT structure [62]–[63]. The Raman spectra of SWCNT-**1** and SWCNT-**2** was acquired by excitation of 514.5 nm laser and a sharp peak corresponding to the G-band at 1590 cm^−1^ was observed (Figure 3). In a control experiment the Raman spectrum of solid SWCNT was recorded (Figure S3). Importantly no change in the spectral nature was observed when the nanotubes were dispersed using amphiphiles **1** and **2**. Also the area under the G-band directly corresponds to the amount of dispersed SWCNT. The comparable area under the G-band in case of both SWCNT-**1** and SWCNT-**2** indicated similar quantity of nanotube dispersion, which was in concurrence with above mentioned quantification.

### In situ synthesis of AgNPs and characterization of nanohybrids

**Figure 4:**
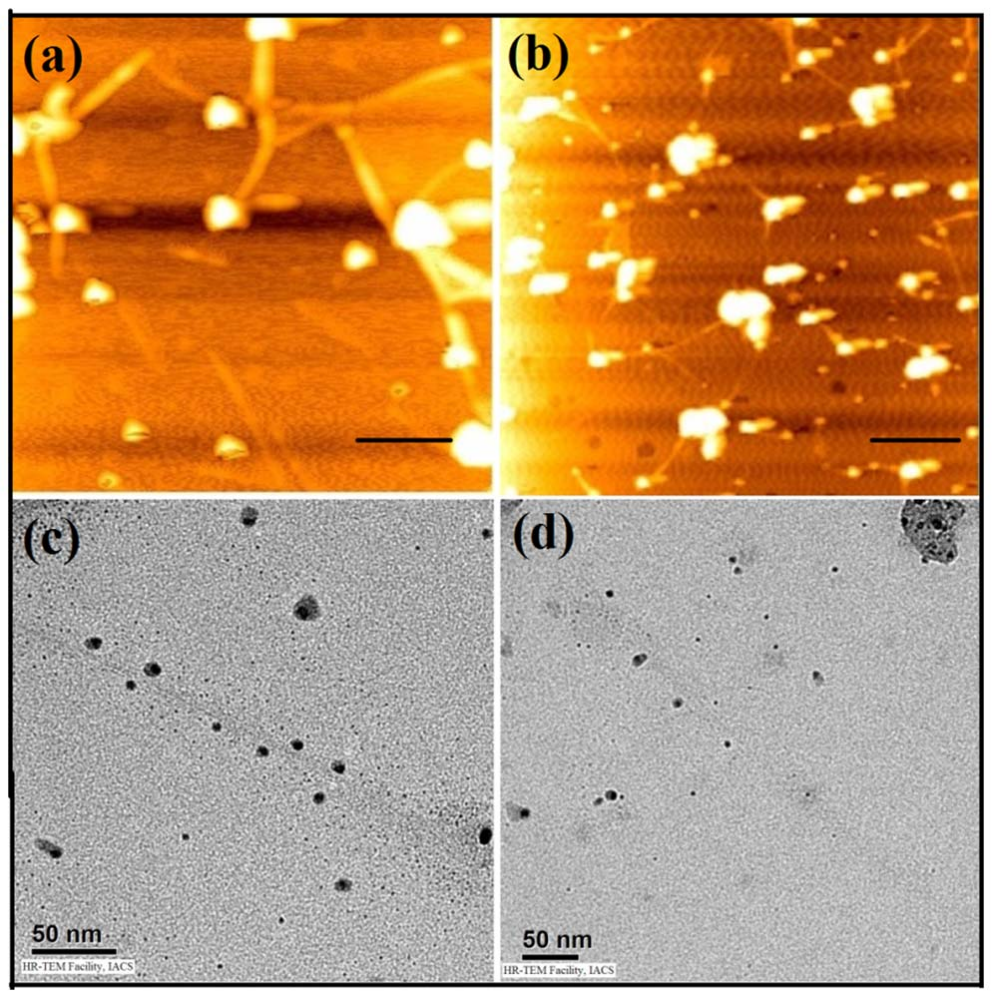
AFM images of (a) SWCNT-1-AgNP, (b) SWCNT-2-AgNP (the scale bars in the AFM images indicate 200 nm) and TEM images of (c) SWCNT-1-AgNP, (d) SWCNT-2-AgNP.

These SWCNT-amphiphile suspensions were subsequently used for the synthesis of AgNPs [58]. A green technique was adopted where AgNPs were synthesized in situ under sunlight from AgNO_3_ at physiological pH. The ratio of AgNO_3_: capping agent (dispersing agents **1** and **2**) was maintained at 1∶10 and the suspensions were exposed to sunlight for 15 min. This photo reduction method does not involve the use of any harmful chemicals [16]–[17]. The formation of AgNPs was monitored by UV-vis spectroscopy. In case of SWCNT-**1**, a surface plasmon resonance (SPR) peak generation was observed after 15 min of sunlight exposure at 425 nm, which gets intensified after 30 min (Figure S4). Similar peak formation due to the synthesis of AgNP was observed at 410 nm in case of SWCNT-**2**. The developed nanoconjugates (SWCNT-**1**-AgNP and SWCNT-**2**-AgNP) were then subjected to microscopic investigations. In the AFM images, AgNPs of 8–10 nm diameters were found to be decorated on the surface of SWCNT-**1** and SWCNT-**2** (Figure 4a,b). Importantly very little amount of unbound nanoparticle was observed. Similarly nanoparticles having a diameter of about 8 to 10 nm were observed on the walls of the nanotubes in the TEM images of SWCNT-**1**-AgNP and SWCNT-**2**-AgNP (Figure 4c,d). Thus the microscopic studies clearly delineated the formation of the nanotube-nanoparticle conjugates. The tryptophan and tyrosine amino acids within the amphiphilic dispersing agents act as capping and stabilizing agent for the synthesized AgNPs that facilitated the binding of the nanoparticles to the nanotube surface [56]–[60]. Interestingly, no loss in colloidal stability of the conjugate was observed when it was kept in dark for a week.

### Quantification of the SWCNT-AgNP nanohybrids

**Figure 5:**
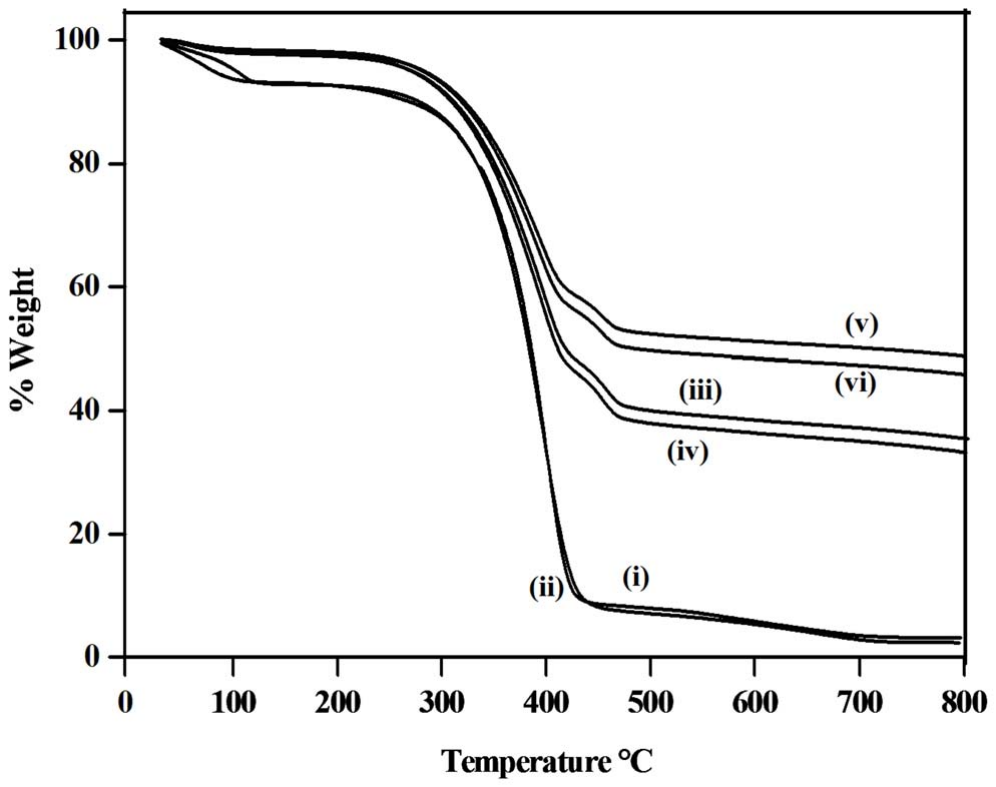
TGA analysis of (i) 1 (ii) 2 (iii) SWCNT-1 (iv) SWCNT-2 (v) SWCNT-1-AgNP and (vi) SWCNT-2-AgNP.

In order to quantify the amount of amphiphile, SWCNT and AgNP present in the nanohybrids, the samples were subjected to thermo gravimetric analysis (TGA). Firstly, SWCNT (30 mg) was sonicated and dispersed using **1** and **2**, respectively, followed by centrifuged at 2500 g to remove heavier bundles. The supernatant was collected and half of the obtained supernatant was taken for in situ synthesis of AgNP. The AgNO_3_ solution was added to this supernatant and AgNP was synthesized in a similar way as described above. Next, all suspensions were centrifuged twice at 45000 rpm to remove excess amphiphiles and unconverted AgNO_3_. The obtained pellets were lyophilized for 6 h to remove trace amount of water. Finally, each conjugate (SWCNT-**1**, SWCNT-**2**, SWCNT-**1**-AgNP and SWCNT-**2**-AgNP) was used for TGA where the samples were heated to 800 °C (Figure 5). As control 10 mg of amphiphiles **1** and **2** were also subjected to the same TGA process of heating. The thermal decomposition pattern of the amphiphiles clearly showed its complete decomposition within 400°C. The TGA plots SWCNT-**1**, SWCNT-**2** indicated the presence of 41 and 39% nanotubes in their respective dispersion. Importantly, the comparison of the TGA plots between the dispersed nanotubes and the AgNP decorated SWCNTs showed that SWCNT-**1**-AgNP contains 41% SWCNT and 14% AgNP while SWCNT-**2**-AgNP contains 39% SWCNT and 12% AgNP. Similarly, AgNP was prepared as control using only amphiphile in the absence of the nanotubes and quantified using TGA (Figure S5). The nanoconjugates were found to contain 30% nanoparticle and 70% amphiphile in both the nanohybrids.

### Antibacterial activity

**Figure 6:**
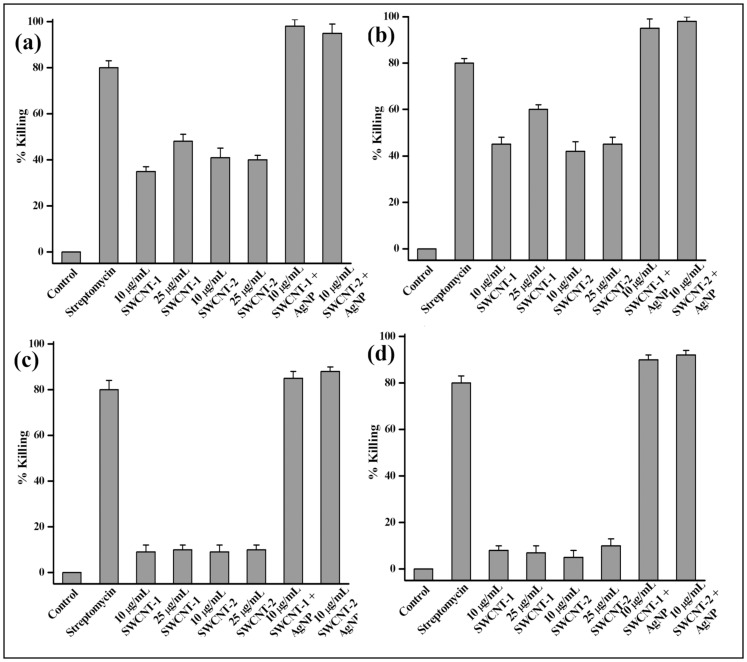
Percentage killing of (a) *B. subtilis*, (b) *M. luteus*, (c) *E. coli* and (d) *K. aragneosa* after 3 h of incubation spread plating for 24 h with the hybrids. Percent killing was determined using colony count method.

Having ensured the formation of the nanotube-nanoparticle hybrids, the conjugates were taken for antibacterial studies. The antibacterial activity was tested against two Gram-positive bacteria *Bacillus* s*ubtilis* (*B*. s*ubtilis*), *Micrococcus luteus* (*M. luteus*) and two Gram-negative bacteria *Escherichia coli* (*E. coli*) and *Klebsiella aerogenes* (*K. aerogenes*). The study was carried out using colony count method. At first the antibacterial activity of the amphiphilic dispersing agents (**1**, **2**) alone was tested against all the said bacterial strains. Both dispersing agents **1** and **2** were inefficient in killing all the four bacterial strains up to 100 µg/mL after 3 h of incubation under shaking condition followed by spread plating for 24 h [36]. Next the antibacterial activity of the SWCNT-dispersing agent was studied against the above mentioned bacterial strains under similar experimental conditions. SWCNT-**1** exhibited moderate bacterial killing efficacy having 35% and 45% killing of *B. subtilis* and *M. luteus*, respectively at 10 µg/mL of the dispersion (Figure 6a,b). This percent killing increased to 48% and 60% when the concentration of the nanotube dispersion was increased to 25 µg/mL. Similarly in case of SWCNT-**2** almost 40% and 45% killing of *B. subtilis* and *M. luteus*, respectively was observed at 25 µg/mL of the dispersion (Figure 6a,b). However when these nanotube dispersions (SWCNT-**1** and SWCNT-**2**) were incubated with Gram-negative bacterial strains *E. coli* and *K. aerogenes* under identical conditions, very poor killing efficacy (<10%) was observed up to 25 µg/mL concentration of both dispersions (Figure 6c,d). The outer membrane of Gram-positive bacteria is composed of peptidoglycan layer comprising of polymeric sugar, and amino acid and phosphoryl substituted teichoic and techuronic acid residues as well as carboxylate groups. However, in case of Gram-negative bacteria the cell membrane is composed of an extra layer of lipopolysaccharide (LPS) and phospholipids in addition to the peptidoglycan layer [36], [64]–[65] This added shielding of the peptidoglycan layer by the presence of LPS might have inhibited the bacteria killing ability of dispersed SWCNTs against Gram-negative bacteria.

To widen the antibacterial spectrum of the nanotube dispersion, antibacterial activity of SWCNT-AgNP conjugates was tested against the above mentioned bacterial strains. AgNPs were synthesized under sunlight as mentioned above and the multimodal bacteria killing efficacy of the nanohybrids was tested against both types of bacterial strains [56]–[58]. Encouragingly, more than 90% of Gram-positive *B. subtilis* and *M. luteus* were killed at 10 µg/mL concentration of SWCNT-**1**-AgNP and SWCNT-**2**-AgNP under similar experimental conditions (Figure 6a,b). Notable improvement in the Gram-positive bacteria killing efficiency was observed for nanotube-nanoparticle hybrids in contrast to that of SWCNT-**1** and SWCNT-**2** dispersions (35–45%) devoid of any AgNPs. Most promisingly, the Gram-negative bacteria which were almost resistant to nanotube dispersions, get efficiently killed by SWCNT-AgNP nanohybrids. SWCNT-**1**-AgNP and SWCNT-**2**-AgNP at 10 µg/mL killed 85–88% *E. coli* and more than 90% *K. aerogenes* under the experimental condition as mentioned above (Figure 6c,d). Thus these newly developed SWCNT-AgNP nanohybrids were equally effective in killing both Gram-positive and Gram-negative bacteria. The killing efficiency of AgNPs synthesized using **1** and **2** in absence of SWCNT was also studied. However, up to 50 µg/mL of AgNP-**1** and AgNP-**2**, negligible killing of (∼10%) was observed against all the four bacterial strains after 3 h of incubation and spread plating for 24 h (Figure S6–S9). Although in previous instances the antibacterial activity was achieved at a comparable concentration of AgNPs, which was probably due to the cumulative effect of the nature of capping agent and the nanoparticle [56]. In the present case, the neutral capping agents do not contribute to the antibacterial activity and the nanoparticles fail to exhibit notable antibacterial activity up to 50 µg/mL. These results clearly delineate the role of the dispersed SWCNTs in increasing the local concentration of AgNPs in vicinity of the bacteria membrane and ultimately facilitating the bacteria killing by the nanoparticles. The intrinsic cell permeability of the nanotubes possibly further aided the interactions of AgNPs along with the nanotube with the bacteria for disintegrating the cell membrane. The presence of nanotube in the newly fabricated SWCNT-AgNP hybrid indeed bolsters the bacteria killing efficiency of the nanoparticles presumably by acting as a cargo transporter. Hence, the antibacterial activity against both Gram-positive and Gram-negative bacteria is achieved by these nanohybrids using the inherent multimodal killing mechanism of AgNPs along with the killing ability of dispersed SWCNTs. Although exact bacteria killing mechanism by AgNPs is not yet understood, AgNPs may act by attacking the phosphorus-containing DNA or interacting with the mitochondria leading to cell death [16]. It may also interact with the sulfur-containing proteins that would hinder the regular cell function. In addition, the release silver ion from AgNPs inside the bacterial cell may lead to bacterial death due to oxidative stress. However due to the lack of stability of the SWCNT in the absence of **1** or **2** it was not possible to study the effect of SWCNT. Similarly in the absence of the dispersing agents no AgNP synthesis takes place hence the effect of SWCNT and AgNP could not be studied separately.

Having studied the antibacterial activity by colony count method, the bacteria killing process was further investigated using live/dead bacterial viability kit. This staining kit is composed of two nucleic acid binding stains, known as SYTO 9 and propidium iodide (PI). Cell membrane permeable SYTO 9 binds to the nucleic acid of both living and dead cells, while propidium iodide can only bind to the nucleic acid of dead cells. Consequently, SYTO 9 labels live bacteria with green fluorescence and PI labels membrane-compromised bacteria with red fluorescence. Gram positive *B. subtilis* and Gram-negative *E. coli* were suspended in 0.9% NaCl and incubated for 3 h with 50 µg/mL of SWCNT-**1** and 10 µg/mL SWCNT-**1**-AgNP. The suspensions were then centrifuged and the supernatant was removed. Subsequently the bacteria were re-suspended in saline containing the live/dead kit and incubated for 30 min. The suspension was then cast onto a slide and visualised under fluorescence microscope. The green fluorescence for untreated *B. subtilis* and *E. coli* indicated that they were alive (Figure S10a,d). Incubation of these bacteria with SWCNT-**1** dispersion exhibited red fluorescence for dead *B. subtilis* (Figure S10b) and green fluorescence for live *E. coli* (Figure S10e). However, predominant presence of dead bacteria (red fluorescence) was observed upon incubation of both *B. subtilis* and *E. coli* with SWCNT-**1**-AgNP (Figure S10c,f). The observed fluorescence microscopic results corroborated well with the data obtained using colony count method.

**Figure 7:**
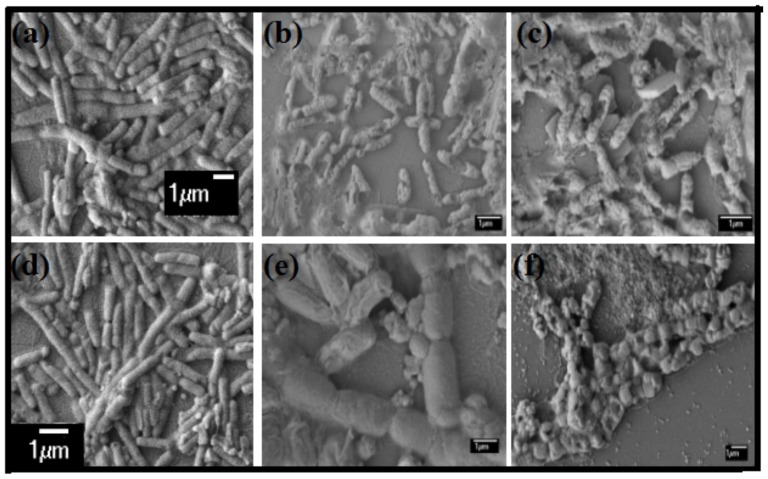
FESEM images of *B. subtilis* incubated with (a) control (b) SWCNT-1 (c) SWCNT-1-AgNP and *E. coli* incubated with (d) control (e) SWCNT-1 and (f) SWCNT-1-AgNP.

To get an insight into the morphology of the bacteria upon incubation with the developed nanohybrids, field emission scanning electron microscopic (FESEM) images of untreated and nanohybrid treated *B. subtilis* and *E. coli* were taken (Figure 7). Both untreated cells showed the normal shape and size of prokaryotic cells (Figure 7a,d). However, upon incubation with 50 µg/mL of SWCNT-**1** dispersion, the cell membrane of Gram-positive *B. subtilis* gets disintegrated (Figure 7b) while cell membrane integrity was mostly unaffected for *E. coli* (Figure 7e). This compromised membrane structure with irregular shape is more predominant in the presence of SWCNT-**1**-AgNP (10 µg/mL) for both *B. subtilis* as well as *E. coli* (Figure 7c,f). The disrupted membrane resulted in the loss of cytoplasmic constituents leading to cell death. The lethal effect of the SWCNT-AgNP nanohybrids was evident in comparison to only SWCNT-amphiphile dispersion. The synergic influence of inherent multimodal killing mechanism of AgNPs along with cell penetrating ability of SWCNTs made these newly developed nanohybrids very efficient in killing both Gram-positive and Gram-negative bacteria.

### Eukaryotic cell viability

**Figure 8:**
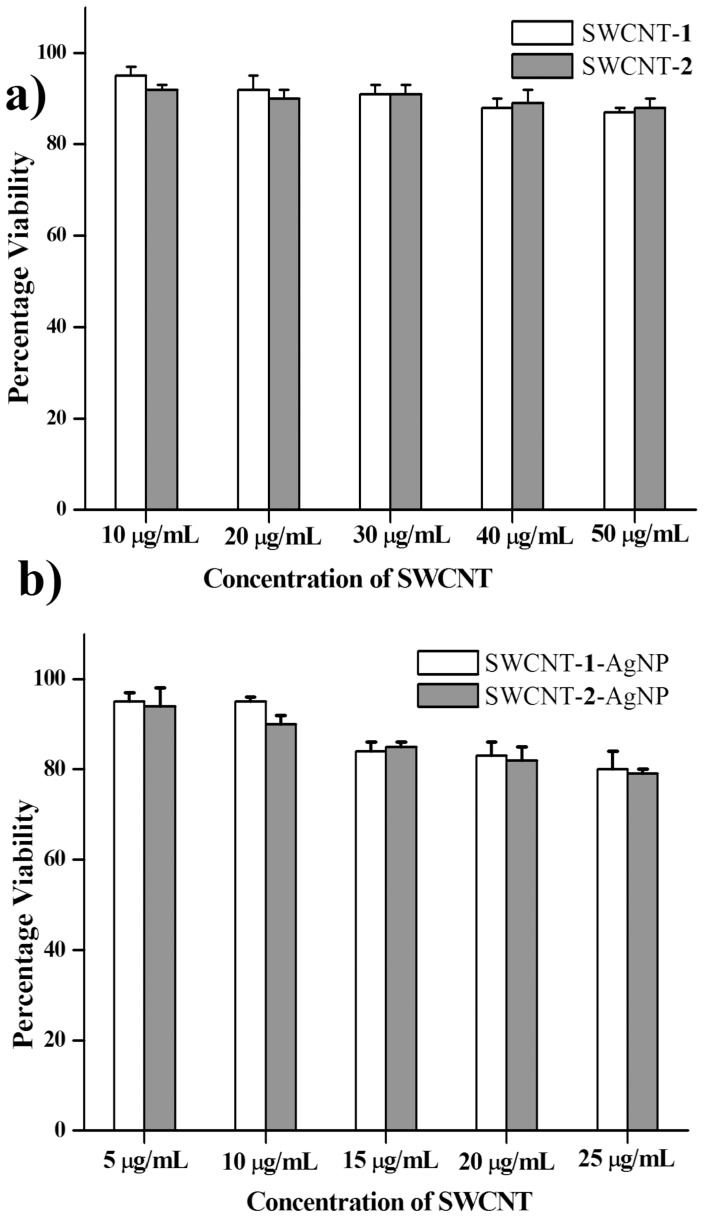
Percentage viability of CHO cells treated with (a) SWCNT-1 and SWCNT-2, (b) SWCNT-1-AgNP and SWCNT-2-AgNP for 24 h.

The biomedicinal application of these newly developed antibacterial nanohybrids will become pertinent only if they exhibit compatibility with eukaryotic cells. The antimicrobial nanohybrids should be lethal to microbes and safe to mammalian cells. The biocompatibility of these nanohybrids to normal eukaryotic cells was studied using MTT based cell viability assay. Initially nanotube dispersions of SWCNT-**1** and SWCNT-**2** at a concentration range of 0-50 µg/mL were incubated with Chinese Hamster Ovarian cells (CHO cells) for 24 h. Encouragingly, up to 50 µg/mL of the nanotube dispersion, 87% and 88% cells remained alive upon incubation with SWCNT-**1** and SWCNT-**2** were used, respectively (Figure 8a). Next the percentage cell viability of CHO cells in the presence of SWCNT-amphiphile-AgNP was checked within a concentration range of 0–25 µg/mL. With varying concentrations of the nanohybrids, promisingly, 80% cells were viable after 24 h of incubation with SWCNT-**1**-AgNP and SWCNT-**2**-AgNP (Figure 8b). Interestingly, within this range of concentration of the nanohybrids ∼90% bacteria undergoes death while >80% eukaryotic cells remain unaffected. This selective killing of bacterial cells in contrast to normal cells probably takes place due to the difference in the constitution of the cell membrane. The bacterial membranes are rich in the thiol (–SH) containing protein present that facilitates its interaction with AgNP of the nanohybrid due to the affinity of silver toward sulphur leading to its death [56]–[58].

### Antibacterial biocompatible film

**Figure 9:**
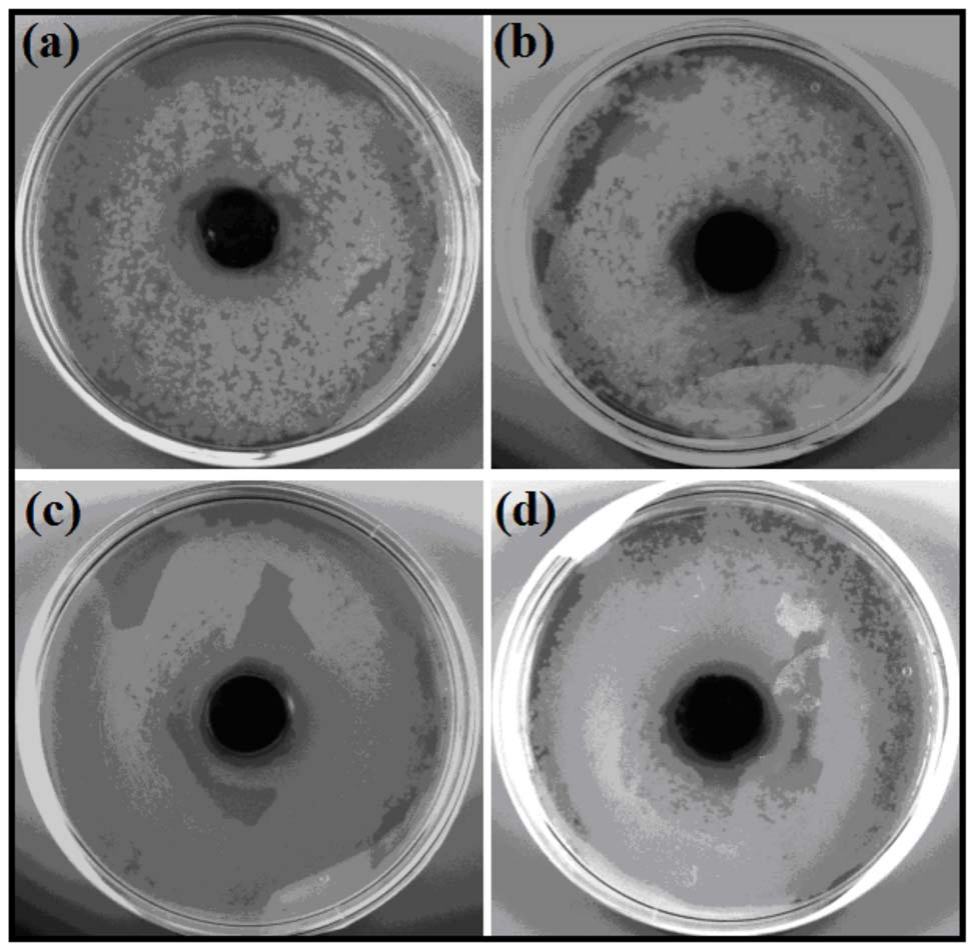
Antibacterial activity of agar-gelatin films against *B. subtilis* (a) SWCNT-1 (b) SWCNT-1-AgNP and *E*. *coli* (c) SWCNT-1 (d) SWCNT-1-AgNP composites.

The antibacterial materials which are also viable to eukaryotic cells are finding surging significance in biomedicine and tissue engineering. In fact, because of the materials induced nosocomial infections in living systems, designing antimicrobial biomaterials for tissue engineering is on the rise. To this end it would be interesting if the present antibacterial nanohybrids could be integrated with known tissue engineering scaffolds to develop superior soft nanocomposites. Agar-gelatin (2∶1) hydrogel cross-linked with glutaraldehyde is well-known matrix that has been used for the proliferation of mice fibroblast cells (NIH3T3) [56], [66]. However, such materials need to have the antibacterial property to be utilized as ideal tissue engineering scaffold. To this objective, the newly developed SWCNT-amphiphile dispersion (50 µg/mL) and SWCNT-amphiphile-AgNP nanohybrids (10 µg/mL) were infused into the agar-gelatin matrices (2∶1). As expected in the absence of either of the dispersion or nanohybrids, normal growth of both *B. subtilis* and *E. coli* was observed. However in the presence of nanohybrid doped material, formation of a clear zone of inhibition was noted (Figure 9, Table S1). This zone of inhibition was less in case of SWCNT-**1** doped polymeric films in both bacterial strains (Figure 9a,c). In fact in case of the Gram-negative strain almost no zone of inhibition could be determined. Importantly, the zone of inhibition increased in the presence of SWCNT-**1**-AgNP nanohybrids (Figure 9b,d, Table S1). Hence, the inclusion of these soft nanohybrids made the tissue engineering scaffold agar-gelatin intrinsically antibacterial.

Now it has become necessary to investigate whether the infusion of the antibacterial soft nanohybrids with the agar-gelatin film affected the cell growth feature on its surface. In this regard we studied the growth and proliferation of normal CHO cells on the surface of these antibacterial polymeric films containing SWCNT-amphiphile (50 µg/mL) and SWCNT-amphiphile-AgNP (10 µg/mL). Cells grown in DMEM were seeded into 24 well plates containing the films and the viability of the cells was studied 24 h post incubation using live/dead viability assay kit (Figure S11). Encouragingly in all the instances bright green spindle shaped cells were observed and almost no red cell was seen, which indicates the healthy nature and growth eukaryotic cells on the agar-gelatin film.

## Conclusion

In summary, biocompatible SWCNT dispersing was prepared and characterized. The SWCNT-dispersion was only lethal towards Gram-positive bacteria. However no bactericidal activity was observed against the Gram-negative bacteria. The dispersions were subsequently used for the synthesis and capping of AgNPs under sunlight. The newly developed AgNP based SWCNT included self assemblies had potent (∼90%) antibacterial activity against both Gram-positive and Gram-negative bacteria. Interestingly the new hybrids showed substantial cell viability towards normal eukaryotic CHO cells after 24 h of incubation. Finally, inclusion of these nanotube-nanoparticle hybrids into well known tissue engineering scaffold agar-gelatin films, made the tissue engineering scaffold intrinsically antibacterial to both Gram-positive and Gram-negative bacteria despite being non-toxic toward mammalian cells. Therefore, the designed soft nanocomposite promises to have immense implications in biomedicine including tissue engineering.

## Materials and Methods

### Materials

Silica gel of 60–120 mesh, L-tryptophan, L-tyrosine, cetyl alcohol, *N*, *N’*-dicyclohexylcarbodiimide (DCC), 4-*N*, *N*-(dimethylamino) pyridine (DMAP), 1-hydroxybenzotriazole (HOBT), succinic anhydride, solvents and all other reagents were procured from SRL, India. Milli-Q water was used throughout the study. Thin layer chromatography was performed on precoated silica gel 60-F_254_ plates of Merck. CDCl_3_, Amberlite Ira-400 chloride ion exchange resins were obtained from Aldrich Chemical Company. Ethylene diaminetetraacetic acid (EDTA) and reagents required to prepare the nutrient broth culture medium like peptone, yeast extract, and agar powder were purchased from Himedia Chemical Company, India. The live/dead baclight bacterial viability kit, live/dead viability/cytotoxicity kit for mammalian cells were purchased from Molecular Probes, Invitrogen Chemical Company. All materials used in the cell culture study such as gelatin, DMEM, heat inactivated FBS, trypsin from porcine pancreas and 3-(4,5-dimethyl-2-thiazolyl)-2,5-diphenyl-2H-tetrazolium bromide (MTT), PEG (M_n_ = 550) were obtained from Sigma Aldrich Chemical Company. ^1^H NMR spectra were recorded on AVANCE 300 MHz (BRUKER) spectrometer. The UV-visible absorption spectra were recorded on a Perkin Elmer Lambda 25 spectrophotometer. Mass Spectrometric (MS) data were acquired by Electron Spray Ionization (ESI) technique on a Q-tof-Micro Quadruple mass spectrophotometer, Micromass. Raman spectra was recorded using laser light (514.5 nm, scattering angle: 908, integration time: 10 s, 20 scans, 75 mW) on a Horiba Jobin Yvon instrument (Model T64000). TEM experiments were performed on a JEOL JEM 2010 high-resolution microscope operated at an accelerating voltage 200 kV. AFM was performed on Veeco, model AP0100 microscope in non-contact mode. Field emission scanning electron microscopy (FESEM) was performed on JEOL-6700F microscope.

### Synthesis of amphiphiles 1 and 2

Briefly, Boc-protected L-amino acids were coupled with *n-*hexadecanol using DCC (1 equivalent) and catalytic amount of DMAP in presence of 1 equivalent of HOBT in dry dichloromethane (DCM). Boc-protected ester was then purified through column chromatography using 60–120 mesh silica gel and acetone/hexane as the eluent. Column purified materials were then subjected to deprotection by trifluoroacetic acid in dry DCM. A drop of anisole was added during the preparation of compound **1** and this was not required for compound **2**. After 2 h of stirring, solvents were removed on a rotary evaporator and the mixture was taken in ethyl acetate. The organic part was washed with 10% aqueous sodium carbonate solution followed by brine to neutrality. The organic part was concentrated to get the corresponding amines. The obtained amine was then refluxed with succinic anhydride in dry DCM for 6 h. It was then washed with brine to remove the unreacted acid anhydride. The acid thus obtained was coupled with PEG unit (having M_n_ = 550) using DCC coupling reaction as mentioned. The coupled product was then purified through column chromatography using 60–120 mesh silica gel and CHCl_3_/MeOH as the eluent. Overall yield was ∼70–80%.

### Characterization of 1 and 2

^1^H NMR of **1** (500 MHz, CDCl_3_, Me_4_Si, 25 °C) δ = 0.82–0.85 (m, 3H), 1.22–1.73 (m, 28H), 2.49–2.70 (m, 4H), 3.25–3.29 (m, 2H), 3.34 (s, 3H), 3.50–3.69 (m, 46H), 3.70–4.10 (m, 2H), 4.23–4.24 (m, 2H), 4.29–4.31 (m, 1H), 7.02–7.52 (m, 5H). MS (ESI): m/z calculated for C_56_H_98_O_17_N_2_: 1070.69; found: 1071.3874 [M + H^+^].

^1^H NMR of **2** (500 MHz, CDCl_3_, Me_4_Si, 25 °C) δ = 0.85–0.88 (m, 3H), 1.25–1.63 (m, 28H), 2.43–2.64 (m, 4H), 2.93–3.18 (m, 2H), 3.37 (s, 3H), 3.45–3.81 (m, 46H), 4.09–4.24 (m, 4H), 4.76–4.78 (m, 1H), 6.29–7.26 (d, 4H). MS (ESI): m/z calculated for C_54_H_97_O_18_N: 1047.67; found: 1048.3489 [M + H^+^].

### Preparation of SWCNT-amphiphile dispersion

To an aqueous solution (4 mL) of the amphiphiles **1** and **2** (2.5 mg/mL) SWCNT (1 mg) was added. The solution was tip-sonicated and bath sonicated for 30 min. The suspension was centrifuged at 2500 g for 90 min to remove the heavy bundles. The amount of the dispersed SWCNTs in the supernatant was calculated from the observed absorbance value at 550 nm that was derived from the previously reported calibration plot of absorbance versus concentration using sodium dodecylbenzene sulfonate (SDBS). The percentage dispersion was calculated as the ratio of the amount of SWCNTs in the dispersion to the amount of SWCNTs initially added.

### Sample preparation for TEM, AFM, Zeta (ζ)-potential and Raman spectroscopy

The SWCNT suspension obtained after centrifugation at 2500 g was ultracentrifuged at 375000 g to remove excess amphiphile and the pellet was re-dispersed in water (SWCNT-**1** and SWCNT-**2**). The aqueous dispersion obtained was used for *ζ*-potential, TEM, AFM and Raman spectroscopy experiments. A drop of the SWCNT suspension was placed on a 300-mesh Cu-coated TEM grid and dried under vacuum for 4 h before taking the image. Similarly in case of AFM studies, a drop of the dispersion was cast on a freshly cleaved mica surface and the samples were air-dried overnight before imaging. The bundle diameter was calculated from the height profile of the nanotubes. From 20 AFM images of a statistical analysis of the bundle diameter and nanotue length were calculated by plotting histogram [49]. Similarly Raman was recorded by excitation of the sample using 514.5 nm laser.

### In situ synthesis of silver nanoparticle (AgNP)

SWCNT (1 mg) was dispersed by sonication in 2.5 mg/mL amphiphile solution as mentioned above. The solution was centrifuged to remove the heavier bundles. To the supernatant AgNO_3_ was added in the ratio 1∶10 (AgNO_3_: amphiphile). The solution was exposed to sunlight for 15 min and the spectra of the samples were taken. A clear peak generation was observed having maxima at 425 nm and 410 nm in case of SWCNT-**1** and SWCNT-**2**, respectively. The peaks intensified upon exposure of the hybrid for 30 min. The prepared samples were centrifuged at 375000 g twice and the supernatant was discarded to remove excess unbound amphiphile and unconverted AgNO_3_.

### TEM and AFM sample preparation of the SWCNT-amphiphile-AgNP nanohybrids

 SWCNT-**1**-AgNP (5 µL) of and SWCNT-**2**-AgNP (5 µL) composites were placed on 300-mesh carbon coated copper grid and freshly cleaved mica and dried under vacuum for 4 h before taking TEM and AFM images.

### Quantification of the SWCNT-amphiphile-AgNP nanohybrids

The samples were then subjected to thermo gravimetric analysis (TGA). SWCNT (30 mg) was dispersed using **1** and **2** as described above. The supernatant was collected and half of the obtained supernatant was taken for the synthesis of AgNP and AgNO_3_ was added in the ratio as mentioned above. Next, all suspensions were centrifuged twice at 375000 g to remove excess surfactant and unconverted AgNO_3_. The obtained pellet was then lyophilized. TGA of each conjugate (SWCNT-**1**, SWCNT-**2**, SWCNT-**1**-AgNP and SWCNT-**2**-AgNP) was done where the samples were heated to 800 °C. The thermal decomposition pattern was monitored. Amphiphiles **1** and **2** as well as AgNP-**1** and AgNP-**2** were also subjected to the same process of heating as control experiments.

### Microorganisms and culture conditions

The antibacterial activity of the nanohybrids was tested against Gram-positive and Gram-negative bacteria. The nutrient broth medium was prepared using peptone (5 g), yeast extract (3 g) in 1 L sterile water. Solid medium for all antibacterial experiments was done by addition of 15 g agar was added in 1 L of the above prepared nutrient broth medium. All the bacteria were purchased from Institute of Microbial Technology, Chandigarh, India. For the experiments, a representative single colony of bacteria was picked up with a wire loop and that loopful was spread on nutrient agar slant to give single colonies and incubated at 37 °C for 24 h. These cultures were diluted as per requirement to give a working concentration in the range of 10^6^–10^9^ colony forming units (cfu)/mL.

### Measurements of antibacterial activity

Antimicrobial activities of SWCNT samples were studied against *Bacillus* s*ubtilis* (*B*. s*ubtilis*), *Micrococcus luteus* (*M. luteus*), *Escherichia coli* (*E. coli*) and *Klebsiella aerogenes* (*K. aerogenes*). Purified SWCNT solid samples were first dispersed in solution of **1** and **2** (2.5 µg/mL) by sonication and centrifugation. The AgNP was synthesized as described above and the excess AgNO_3_ and surfactant was removed by ultra centrifugation at 37500 g. A 10 mL portion of dispersion was incubated with 1 mL of bacterial suspensions (10^6^–10^7^ cfu/mL) for 2 h under shaking at 37 °C. The antimicrobial evaluations were carried out by a colony forming count method. For the colony forming count method, 100 µL serial 10-fold dilutions with saline solution was spread onto agar plates and left to grow overnight at 37 °C. Colonies were counted and compared with control plates to calculate percentage killing. All treatments were prepared in duplicate and repeated on at least three separate occasions.

### FESEM of nanocomposite treated bacteria

The morphological changes of B. subtilis and E. coli were investigated by field emission scanning electron microscopy (FESEM). Treated bacterial suspensions were concentrated by centrifugation at 5000 rpm and then the cells were dropped on a glass slide to dry at room temperature. The dried samples were sputter coated with gold for FESEM imaging.

### Fluorescence microscopic study for bacteria

To examine bacterial cell viability live/dead bacterial kit was used. The kit consists of a mixture of SYTO 9 and propidium iodide which are two nucleic-acid binding stains. *B. subtilis* (5×10^6^–7.5×10^6^ cfu/mL) and *E. coli* (3.75×10^7^–7.5×10^7^ cfu/mL) cells (1 mL) were treated with SWCNT-**1** (50 µg/mL) and SWCNT-**1**-AgNP (10 µg/mL) and also untreated cells were taken in centrifuge tube as control. The mixtures were centrifuged at 5,000 rpm for 5 min. Then, media was removed and the cells were re-dispersed in 0.9 wt% saline. Finally, the dye mixture was added and incubated in dark at room temperature for 15–20 min. After incubation, 5 µL of the solution mixture was mounted over microscope slides, air-dried and viewed under the microscope (BX61, Olympus) (ex/em ∼495 nm/515 nm for SYTO 9 and ex/em ∼ 495 nm/635 nm for propidium iodide).

### Cell cultures

Mouse fibroblast NIH3T3 cells were obtained from National Center for Cell Science (NCCS), Pune (India), and cultured in DMEM medium containing 10% FBS, 100 mg/L streptomycin and 100 IU/mL penicillin. Cells were grown in 25 mL cell culture flask and incubated at 37 °C in a humidified atmosphere of 5% CO_2_ to approximately 70–80% confluence. Fresh media was added after every 2–3 days and subculture was performed every 7 days. Next, the adherent cells were detached from the surface of the culture flask by trypsinization and seeded into plates for cytotoxicity assay and imaging experiments.

### Cytotoxicity assay

Cytotoxicity of nanocomposites SWCNT-**1**, SWCNT-**2**, SWCNT-**1**-AgNP and SWCNT-**2**-AgNP were assessed by the microculture MTT reduction assay [67]. This assay is based on the reduction of a soluble tetrazolium salt by mitochondrial dehydrogenase of the viable cells to water insoluble coloured product, formazan. The amount of formazan formed can be measured spectrophotometrically after dissolution of the dye in DMSO. The activity of the enzyme and the amount of the formazan produced is proportional to the number of cells alive. Cells were seeded at a density 15,000 cells per well in a 96-well microtiter plate for 18–24 h before the assay. Stock solutions of all the composites were prepared in water. Sequential dilutions of these stock solutions were done during the experiment to vary the concentrations of SWCNT-amphiphile (5–50 µg/mL) and SWCNT-amphiphile-AgNP (5–25 µg/mL) in the microtiter plate. The cells were incubated for 4 h at 37 °C under 5% CO_2_. Then, 15 µL MTT stock solution (5 mg/mL) in phosphate buffer saline was added to the above mixture and further incubated for 4 h. The precipitated formazan was dissolved thoroughly in DMSO and absorbance at 570 nm was measured using BioTek Elisa Reader. The number of surviving cells were expressed as percent viability  =  (A_570_(treated cells)-background/A_570_(untreated cells)-background) ×100.

### Preparation of agar-gelatin gel film

The mixture (1 wt%) of agar and gelatin in the weight ratio 2∶1 was taken in PBS (pH 7.4) and dissolved by heating. In case of SWCNT and SWCNT-amphiphile-AgNP containing film preparation, nanocomposite solution was added to this homogeneous mixture of agar-gelatin so that 50 µg/mL of SWCNT-**1** and 10 µg/mL of SWCNT-**1**-AgNP could be attained. The components were crosslinked by the addition of glutaraldehyde (0.15 wt%) to the hot solution. Each solution (1 mL) was poured into 24-wells tissue culture flask and left at room temperature. Then the plates containing gels were dried in the oven for 12 h at 50 °C to form thin films. These prepared films were treated with 0.1 mM glycine for 1 h to block the remaining aldehyde group. The films were then washed several times with Milli-Q water to remove excess glycine and then with PBS to neutralize the surface. These films were then again dried and UV sterilized overnight and kept in sterile vacuum desiccators for further experiment.

### Antibacterial activity of agar-gelatin films

The antibacterial activity of agar-gelatin films against *B*. *Subtilis* and *E*. *coli* was followed in nutrient agar plates. The agar-gelatin films with and without the nanohybrids was placed on the middle of the freshly prepared agar plates. For this, each bacterium was cultured on nutrient agar slant at 37 °C for 24 h. These overnight cultures of bacteria were diluted as required to get a concentration of ∼10^5^ cfu/mL. This bacteria containing solution (1 mL) was added to nutrient agar plate. The plates were then incubated for 24 hours at 37 °C. The bacteria killing ability of the films was followed by measuring the zone of inhibition in the agar plate.

### Biocompatibility of agar-gelatin films

The NIH3T3 cell attachment studies were done on control well (24 well plate) without any film and on agar-gelatin films with SWCNT-**1**, SWCNT-**2**, SWCNT-**1**-AgNP and SWCNT-**2**-AgNP. Films were soaked with cell culture media for 3 h. After 3 h the media was removed and 5×10^5^ cells were seeded in each well with 1 mL media. The cells were incubated for 24 h in a 5% CO_2_ atmosphere. After 24 h the adherent cells were washed with PBS and cell viability was examined under a fluorescence microscope using the live/dead viability/cytotoxicity kit for mammalian cells. The kit contains Calcein AM and Ethidium homodimer-1(EthD-1). The supplied 2 mM EthD-1 stock solution (4 µL) and 4 mM calcein AM stock solution (1 µL) was added to 2 mL of sterile, tissue culture-grade PBS and the mixture was vortexed to ensure thorough mixing. The final stock solution (500 µL) of calcein AM (2 µM) and EthD-1 (4 µM) was then added directly to each well containing NIH3T3 cells. After 30 min incubation in darkness, the cells were viewed under Olympus IX51 inverted microscope (ex/em ∼495 nm/515 nm for calcein AM and ex/em ∼ 495 nm/635 nm) for EthD-1.

## Supporting Information

Figure S1 **Histogram for the determination of average bundle diameter of the nanotubes.**
(TIF)

Figure S2 **Histogram for the determination of average length of the nanotubes.**
(TIF)

Figure S3 **Raman spectra of pristine SWCNT.**
(TIF)

Figure S4 **Time dependent UV-vis spectra of synthesized AgNP by (a) SWCNT-1 and (b) SWCNT-2 after (i) 15 min and (ii) 30 min.**
(TIF)

Figure S5 **TGA analysis of AgNP-1 and AgNP-2.**
(TIF)

Figure S6 **Percentage killing of** ***B. subtillis*** **after 3 h of incubation and spread plating for 24 h with the varying concentration of AgNP capped with 1 and 2.** Percent killing was determined using colony count method.
(TIF)

Figure S7 **Percentage killing of** ***M. leuteus*** **after 3 h of incubation and spread plating for 24 h with the varying concentration of AgNP capped with 1 and 2.** Percent killing was determined using colony count method.
(TIF)

Figure S8 **Percentage killing of** ***E. coli*** **after 3 h of incubation and spread plating for 24 h with the varying concentration of AgNP capped with 1 and 2.** Percent killing was determined using colony count method.
(TIF)

Figure S9 **Percentage killing of** ***K. aragneosa*** **after 3 h of incubation and spread plating for 24 h with the varying concentration of AgNP capped with 1 and 2.** Percent killing was determined using colony count method.
(TIF)

Figure S10 **Fluorescence micrographs of** ***B. subtilis*** **incubated with (a) control (b) SWCNT-1 (c) SWCNT-1-AgNP and** ***E. coli*** **incubated with (d) control (e) SWCNT-1 and (f) SWCNT-1-AgNP followed by incubation with live/dead kit.**
(TIF)

Figure S11 **Live dead images of CHO cells grown on agar gelatin films containing (a,b) SWCNT-1 (c,d) SWCNT-1-AgNP (e,f) SWCNT-2 and (g,h) SWCNT-2-AgNP.**
(TIF)

Table S1 **Zone of Inhibition (mm) for Agar-Gelatin Films Containing Soft Nanohybrids.**
(DOC)
