# Supplementary figures and images for: Fabrication of SWCNT-Ag Nanoparticle Hybrid Included Self-Assemblies for Antibacterial Applications
Source: PLoS One. 2014 Sep 5;9(9):e106775. doi: 10.1371/journal.pone.0106775 (PMC4159779; doi:10.1371/journal.pone.0106775)

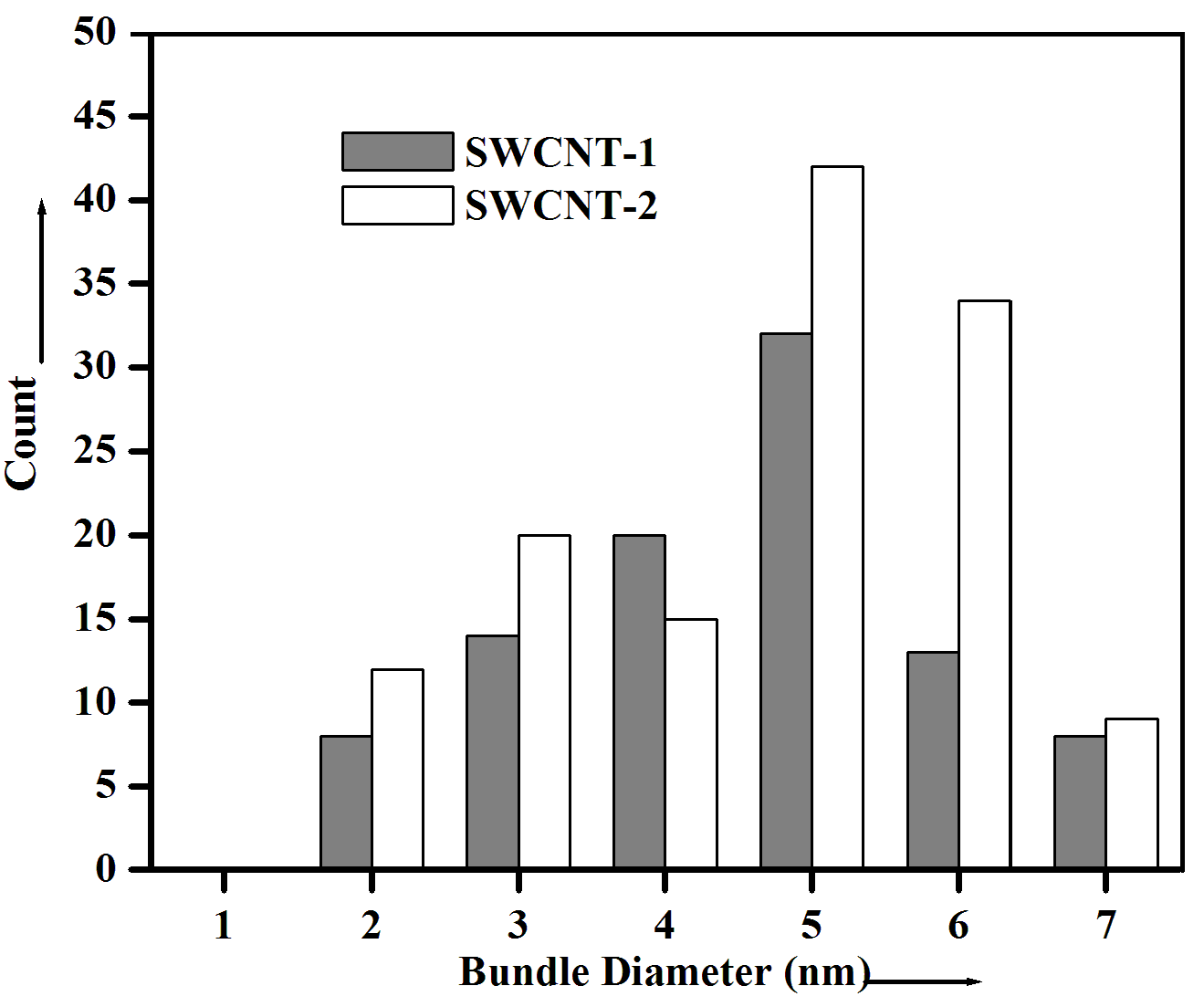

Supplement: Figure S1 — Histogram for the determination of average bundle diameter of the nanotubes. (TIF) [file pone.0106775.s001.tif]

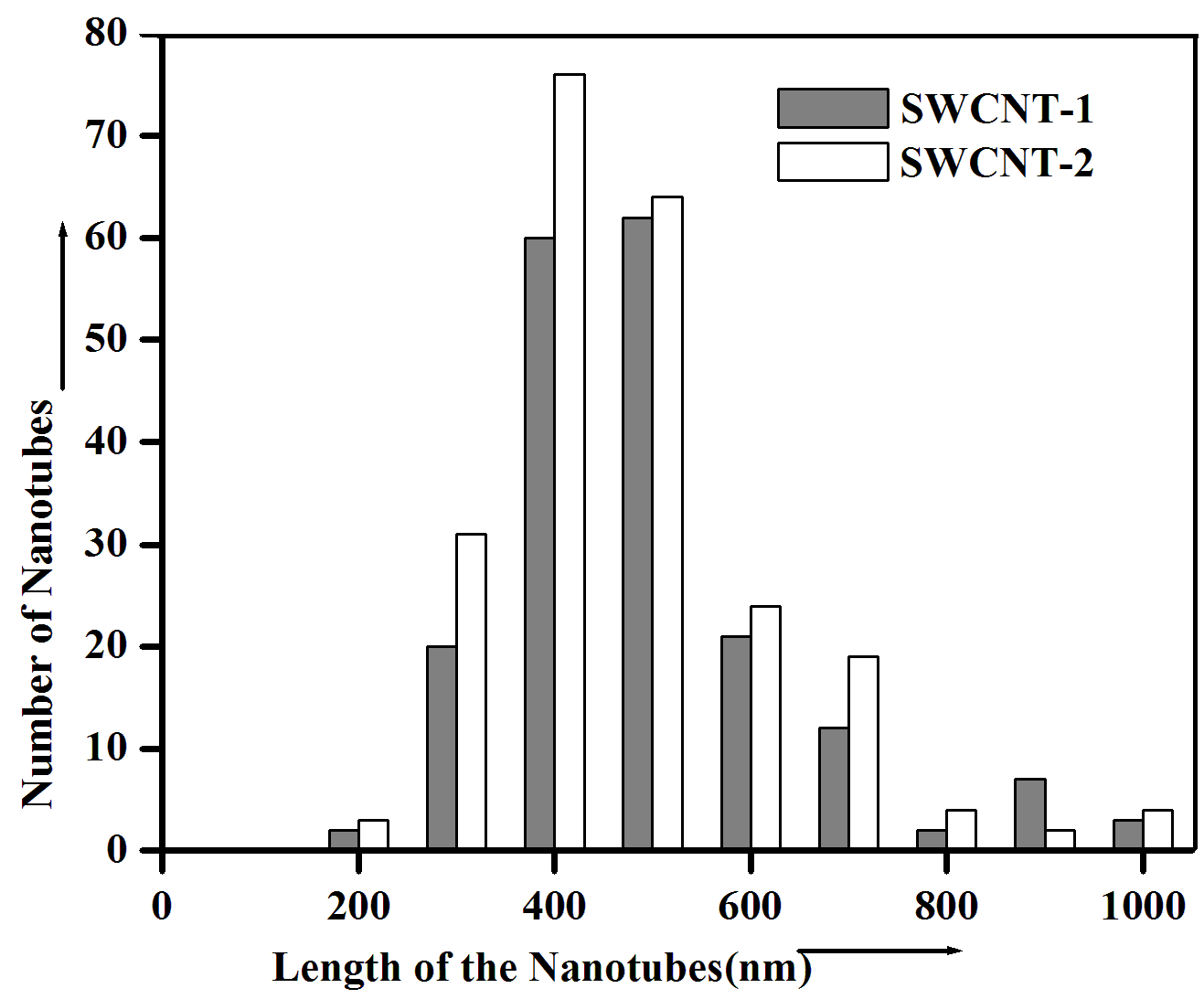

Supplement: Figure S2 — Histogram for the determination of average length of the nanotubes. (TIF) [file pone.0106775.s002.tif]

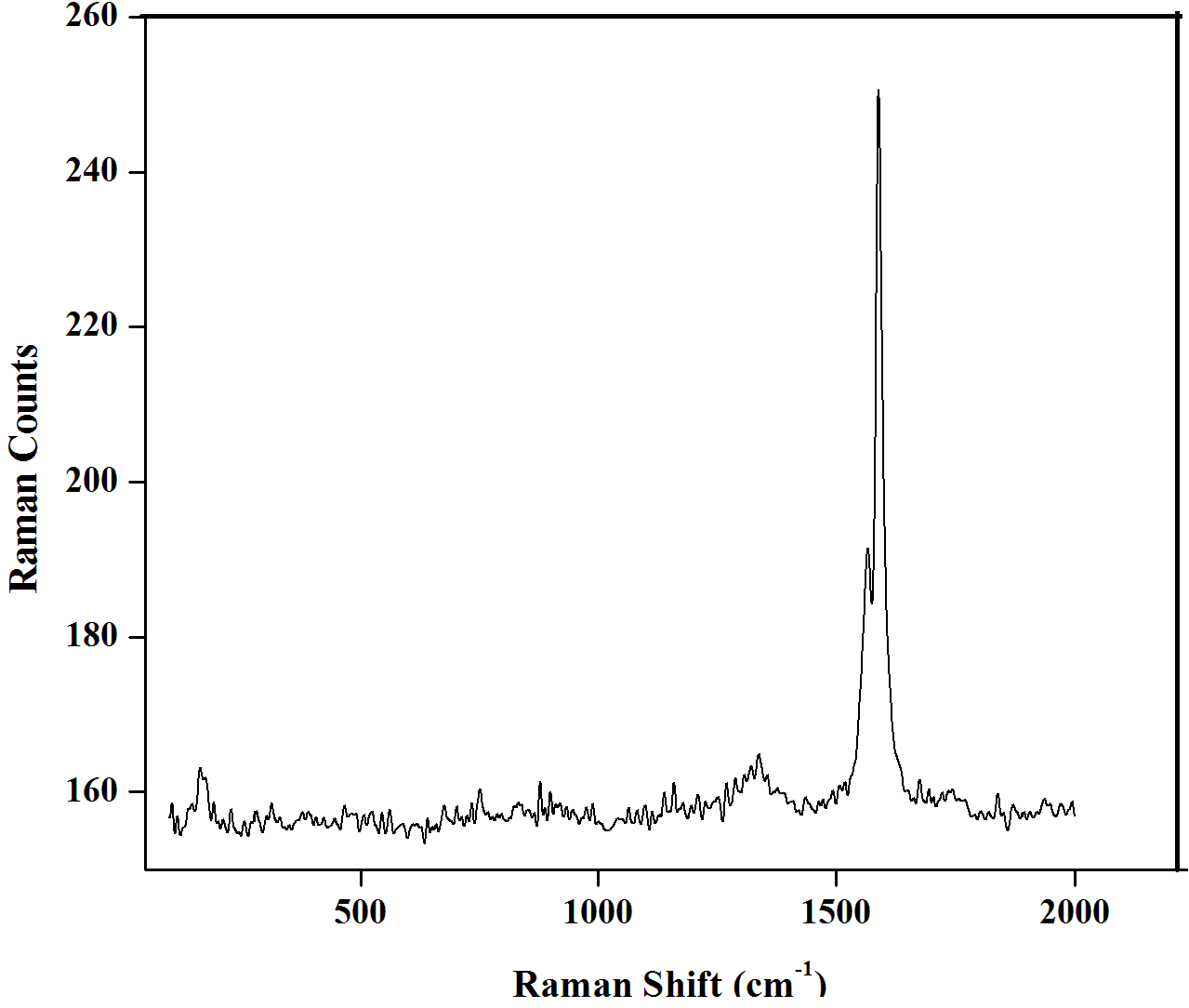

Supplement: Figure S3 — Raman spectra of pristine SWCNT. (TIF) [file pone.0106775.s003.tif]

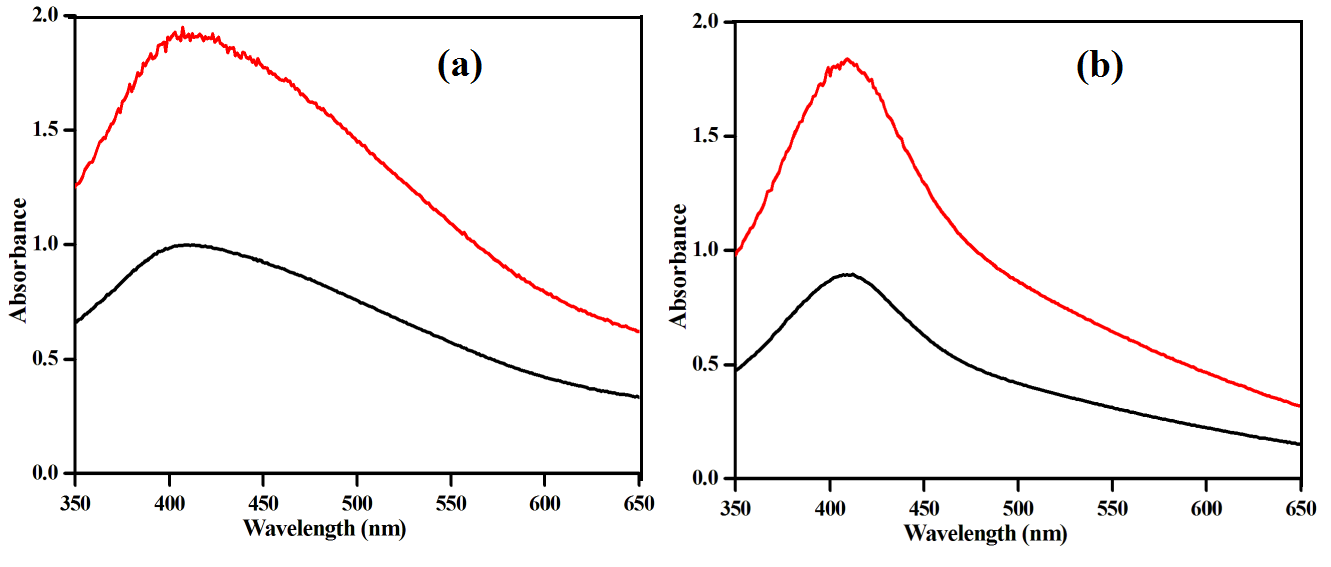

Supplement: Figure S4 — Time dependent UV-vis spectra of synthesized AgNP by (a) SWCNT-1 and (b) SWCNT-2 after (i) 15 min and (ii) 30 min. (TIF) [file pone.0106775.s004.tif]

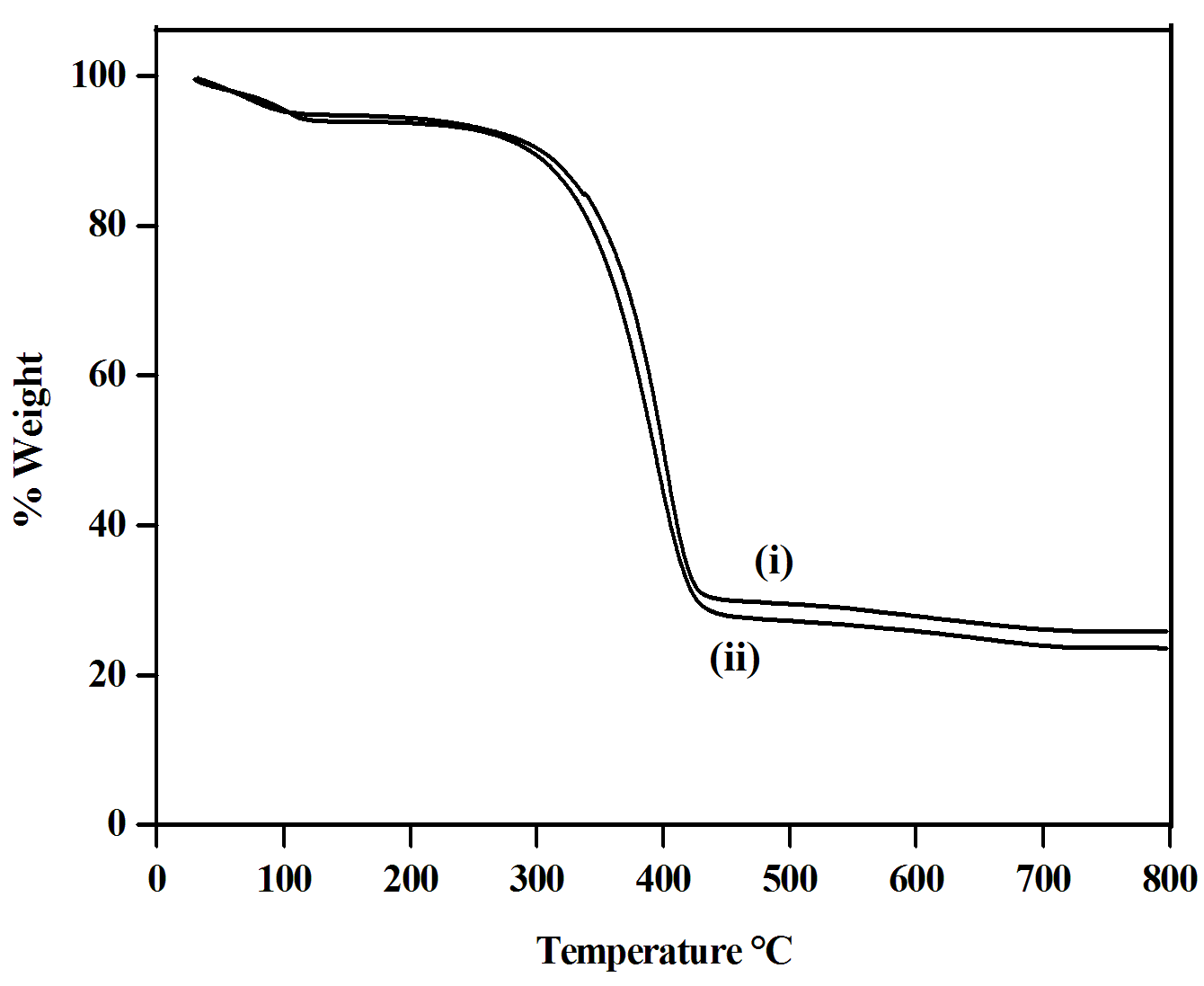

Supplement: Figure S5 — TGA analysis of AgNP-1 and AgNP-2. (TIF) [file pone.0106775.s005.tif]

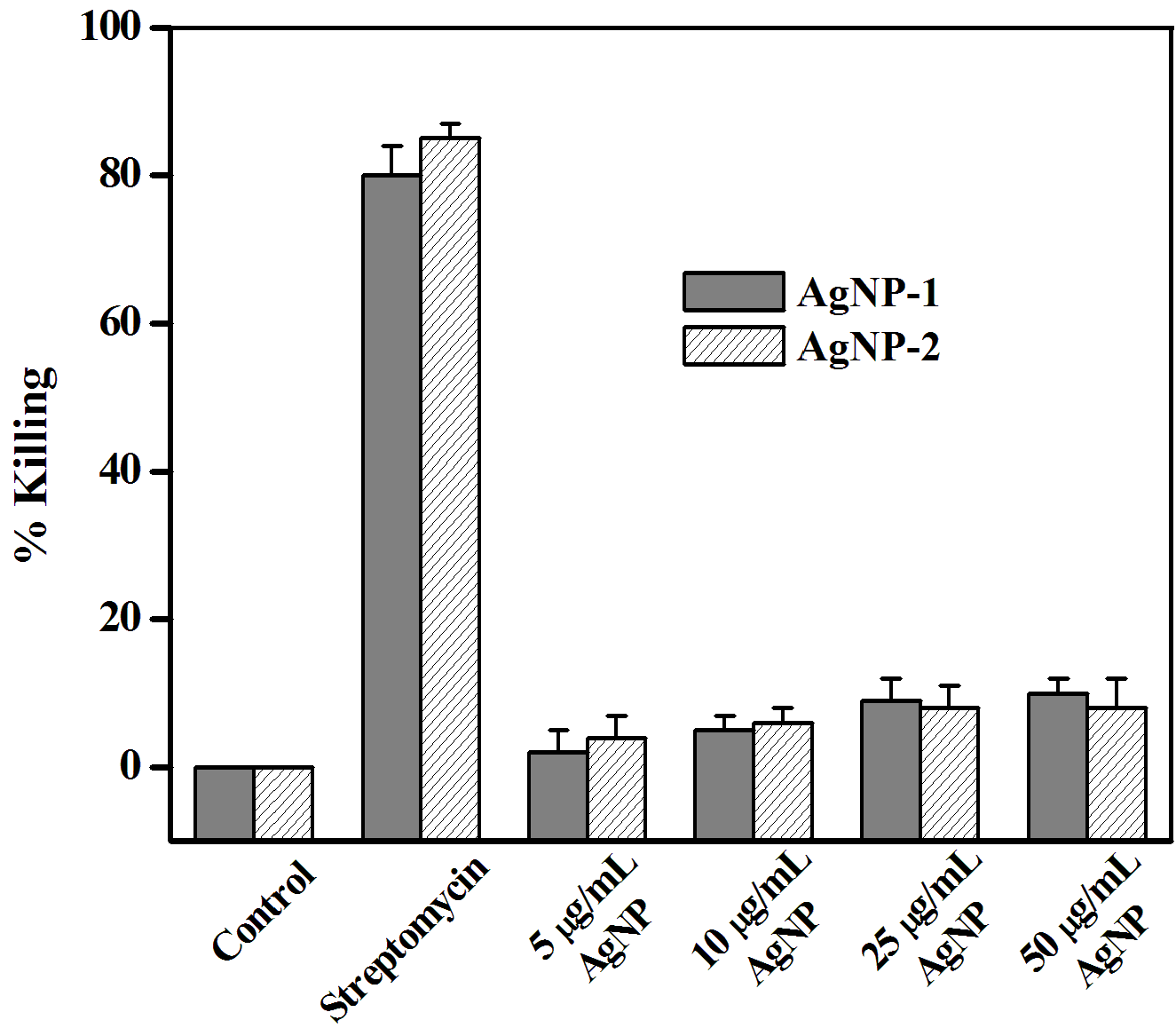

Supplement: Figure S6 — Percentage killing of B. subtillis after 3 h of incubation and spread plating for 24 h with the varying concentration of AgNP capped with 1 and 2. Percent killing was determined using colony count method. (TIF) [file pone.0106775.s006.tif]

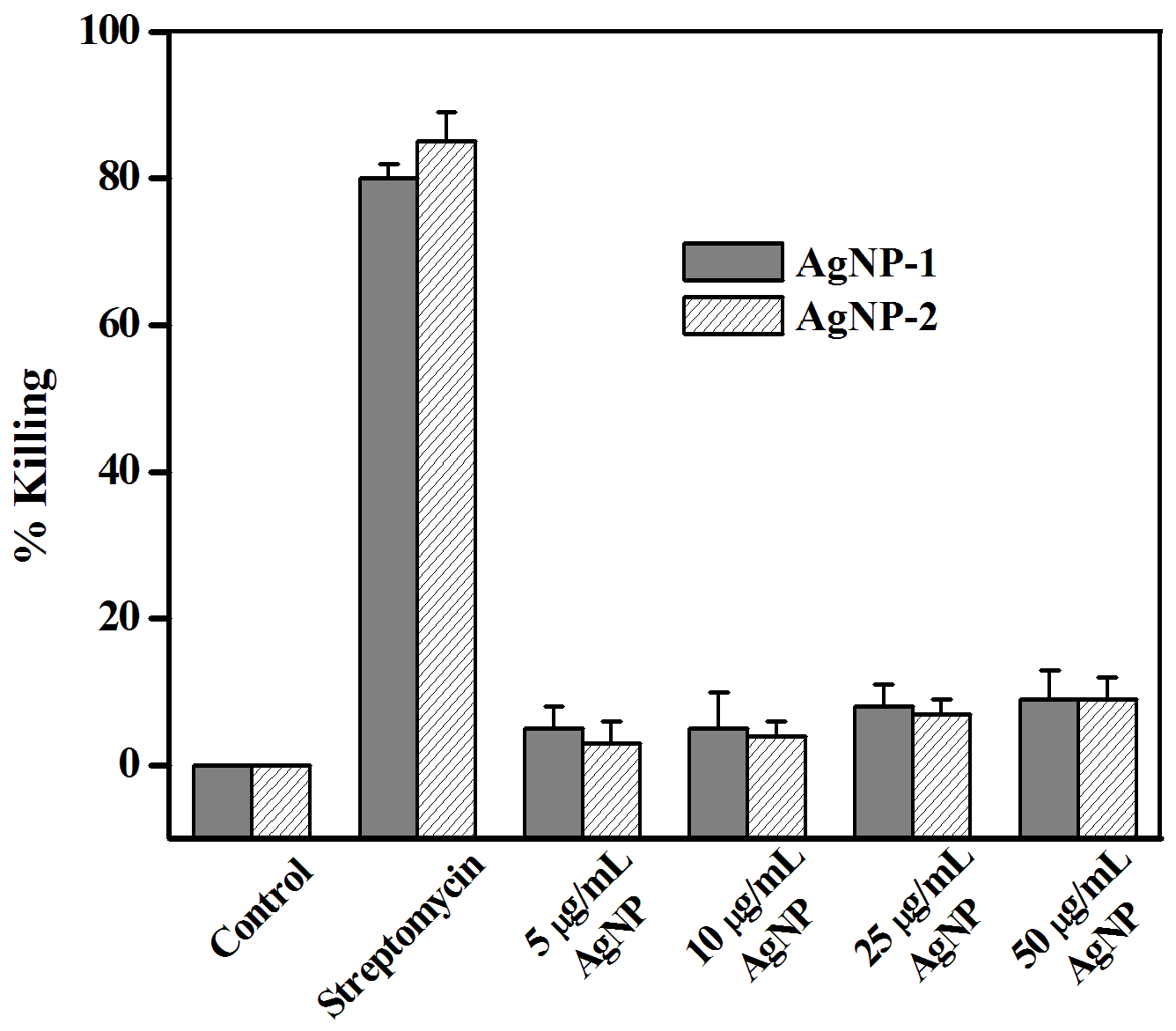

Supplement: Figure S7 — Percentage killing of M. leuteus after 3 h of incubation and spread plating for 24 h with the varying concentration of AgNP capped with 1 and 2. Percent killing was determined using colony count method. (TIF) [file pone.0106775.s007.tif]

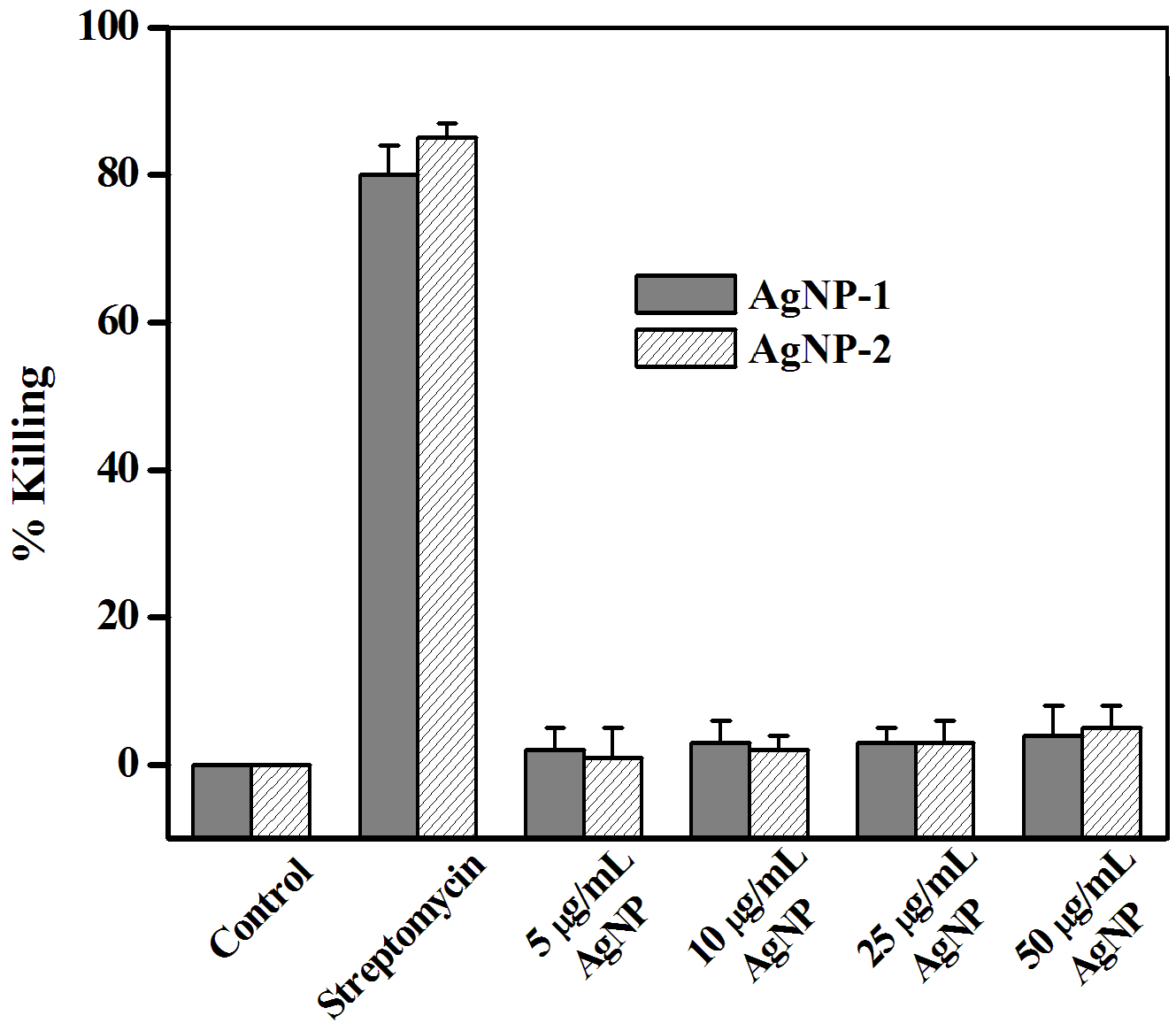

Supplement: Figure S8 — Percentage killing of E. coli after 3 h of incubation and spread plating for 24 h with the varying concentration of AgNP capped with 1 and 2. Percent killing was determined using colony count method. (TIF) [file pone.0106775.s008.tif]

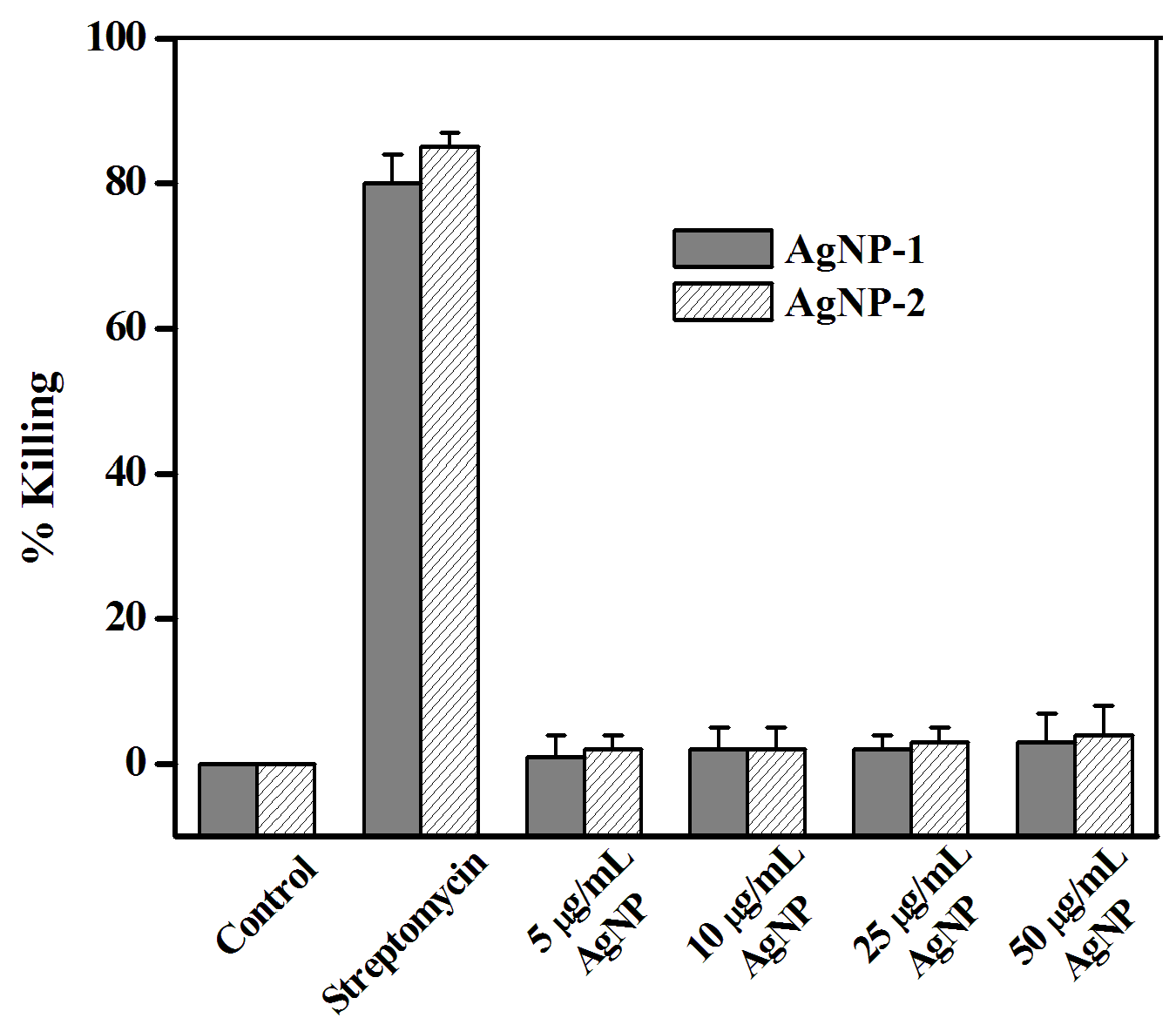

Supplement: Figure S9 — Percentage killing of K. aragneosa after 3 h of incubation and spread plating for 24 h with the varying concentration of AgNP capped with 1 and 2. Percent killing was determined using colony count method. (TIF) [file pone.0106775.s009.tif]

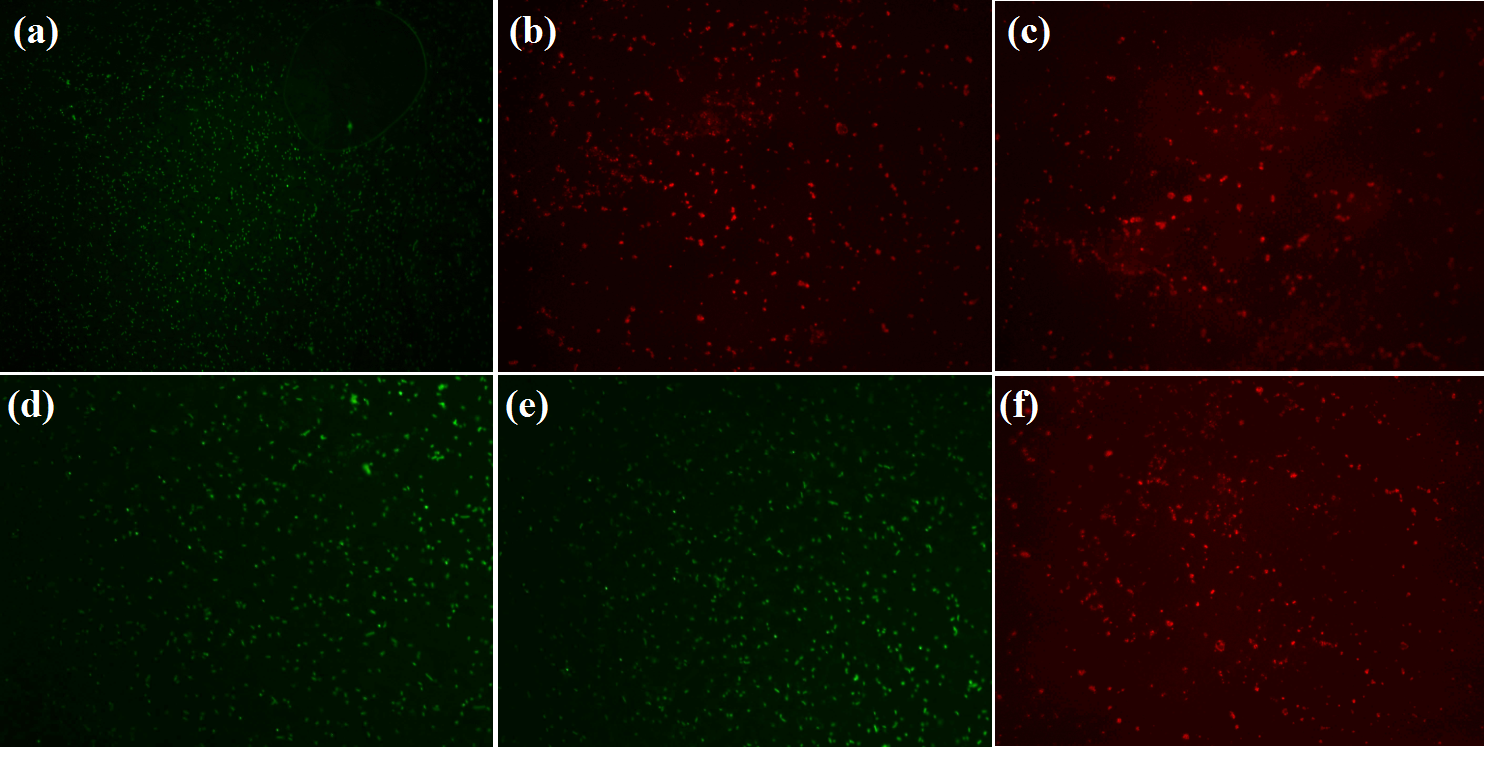

Supplement: Figure S10 — Fluorescence micrographs of B. subtilis incubated with (a) control (b) SWCNT-1 (c) SWCNT-1-AgNP and E. coli incubated with (d) control (e) SWCNT-1 and (f) SWCNT-1-AgNP followed by incubation with live/dead kit. (TIF) [file pone.0106775.s010.tif]

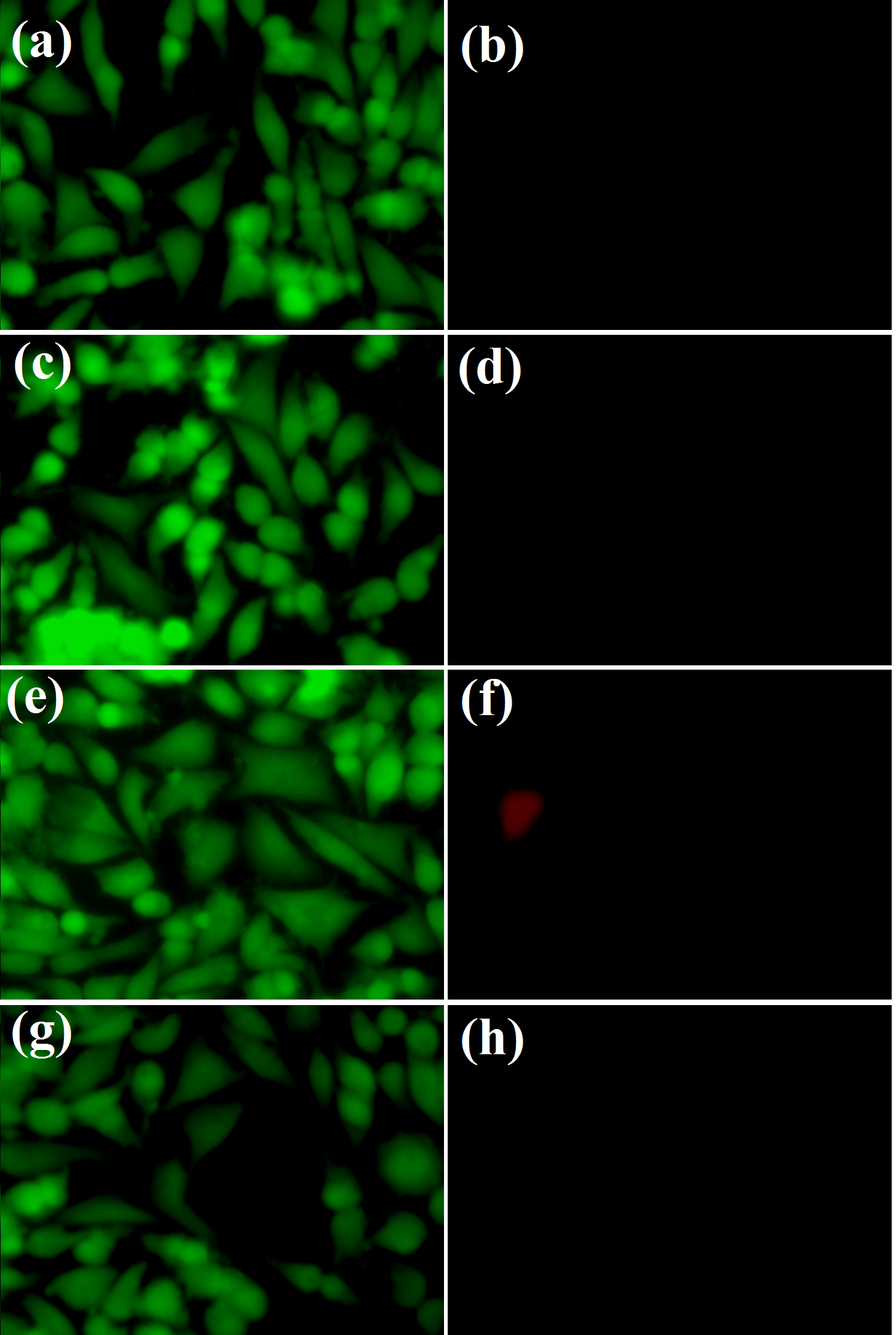

Supplement: Figure S11 — Live dead images of CHO cells grown on agar gelatin films containing (a,b) SWCNT-1 (c,d) SWCNT-1-AgNP (e,f) SWCNT-2 and (g,h) SWCNT-2-AgNP. (TIF) [file pone.0106775.s011.tif]
