# Supplementary material for: Fabrication of SWCNT-Ag Nanoparticle Hybrid Included Self-Assemblies for Antibacterial Applications
Source: PLoS One. 2014 Sep 5;9(9):e106775. doi: 10.1371/journal.pone.0106775 (PMC4159779; doi:10.1371/journal.pone.0106775)
Supplement: Table S1 — Zone of Inhibition (mm) for Agar-Gelatin Films Containing Soft Nanohybrids. (DOC) [file pone.0106775.s012.doc]

**Table S1.**

| **Nanoconjugates** | ***B*. *Subtilis*** | ***E*. *coli*** |
| --- | --- | --- |
| SWCNT-**1** | 2.0 | Undetermined |
| SWCNT-**1**-AgNP | 4.0 | 3.5 |
